# Supplementary material for: Machine learning prediction of the degree of food processing
Source: Nat Commun. 2023 Apr 21;14:2312. doi: 10.1038/s41467-023-37457-1 (PMC10121643; doi:10.1038/s41467-023-37457-1)
Supplement: Supplementary file 1 — Supplementary Information [file 41467_2023_37457_MOESM1_ESM.pdf]

Supplementary Materials

Machine Learning Prediction of the Degree of Food Processing

Giulia Menichetti[1,2], Babak Ravandi[2], Dariush Mozaffarian[3], Albert-László Barabási[2,4,5,†]

[1]Channing Division of Network Medicine, Department of Medicine, Brigham and Women's Hospital, Harvard Medical School, Boston, USA
[2]Network Science Institute and Department of Physics, Northeastern University, Boston, USA
[3]Tufts Friedman School of Nutrition Science and Policy, Tufts School of Medicine and Medical Center, Boston, USA
[4]Department of Network and Data Science, Central European University, Budapest, Hungary
[5]Department of Medicine, Brigham and Women's Hospital, Harvard Medical School, Boston, USA
†Corresponding author. e-mail: a.barabasi@northeastern.edu

Contents

1 Training Dataset **3**

1.1 Food Composition Databases 3

1.2 FNDDS 2009-2010 4

1.3 NOVA Manual Classification Coverage 6

1.4 Nutrient Panels 7

1.5 Hierarchical Clustering of Foods according to Nutrients 9

2 Random Forest Classifier FoodProX and Food Processing Score $FPro$ **11**

2.1 Random Forest Classifier 11

2.2 Feature Importance 12

2.3 Food Processing Score $FPro$ 14

2.4 Validation of $FPro$ in Different FNDDS Editions 17

2.5 Case Study on Post Cereals. 18

2.6 Source of Food 18

3 Individual Diet Processing Scores $iFPro$ and Exposome **20**

3.1 Individual Processing Score $iFPro$ 20

3.2 Population Characteristics 20

3.3 Correlation between $iFPro_{WG}$, $iFPro_{WC}$, and HEI-15 21

3.4 Water Consumption in NHANES 23

3.5 Relation between $iFPro_{WC}$ and WWEIA Food Categories 23

4 Environment-Wide Association Study **25**

5 Food Substitution **34**

6 Open Food Facts **35**

7 Data Quality and Future Directions **37**

1 Training Dataset

1.1 Food Composition Databases

The food supply, representing the full inventory of all foods available for human consumption, along with their nutritional content, plays an important role in determining an individual's nutrient exposure. Nutritional information is captured by food databases, collections of nutrient measurements for extensive samples of the food supply. Here we define as nutrients all chemicals cataloged by food databases, whether they refer to unique chemicals, like vitamin C, or aggregate measures, like total fat or total sugar. Additionally, all major nutrient databases include calories, measuring how much energy our body could get from eating or drinking the selected product. Which foods and which nutrients to report is strictly dependent on the database considered. For instance, USDA SR Legacy, the authoritative source of food composition data in the United States contains 7,793 food items with variable nutrient resolution, from a minimum of 8 nutrients, up to 138 (Figure S1) [2]. In comparison, USDA FNDDS, designed for the epidemiological analysis of dietary intake data collected by the National Health and Nutrition Examination Survey (NHANES), reports 65 to 102 nutrients for all foods, depending on the edition, containing no missing nutrient values (Figure S1) [3, 4].

The nutrient resolution available to consumers is significantly lower: the Food and Drug Administration (FDA) mandates the listing of 14 nutrients on the nutrition facts label, from saturated and trans fat, to sodium and vitamin C [1] An updated nutrition facts label was finalized in 2016, removing vitamins A and C, but listing added sugars, vitamin D, and potassium. However, the compliance deadline for certain food categories was extended to July 2021, and for the majority of the data describing branded products, we observed a significantly higher coverage of the nutrition facts prior to 2016.

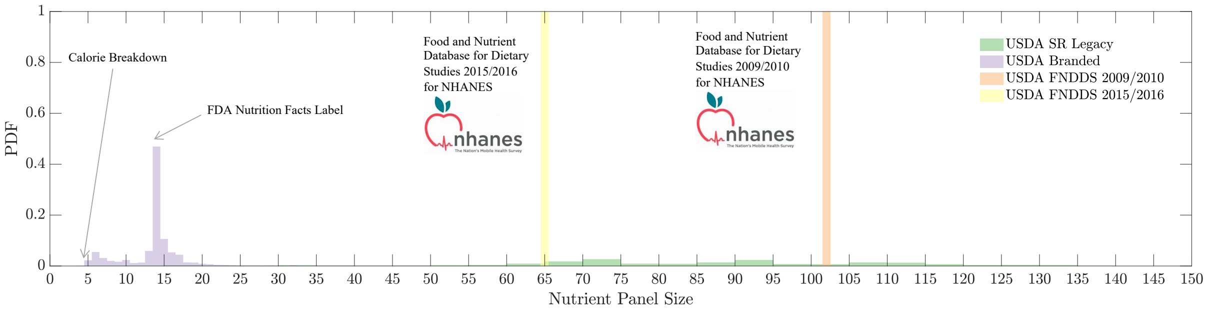

Figure S1: **Nutrient panel resolution for different food databases**. To fully capture the nutrient alterations caused by food processing, we need access to the nutrient information characterizing each food in the food supply. The resolution available for branded products sold in grocery stores is very limited, frequently less than what is required by FDA nutrition facts [1].

1.2 FNDDS 2009-2010

The Food and Nutrient Database for Dietary Studies (FNDDS) is designed by the USDA to provide food composition data (e.g., the amount of vitamin C per 100 g of a selected ingredient) for foods and beverages reported in the dietary component of the National Health and Nutrition Examination Survey (NHANES), a biannual cross-sectional survey of the US Population conducted by Center for Disease Control and Prevention (CDC) to monitor the health of Americans. FNDDS is derived by combining the food items provided in the USDA National Nutrient Database for Standard Reference (SR). In other words, each item in FNDDS is related to one or more foods in SR, reported as ingredients in FNDDS. Differently from SR, designed for the dissemination of food composition data, FNDDS's goal is to enable the analysis of dietary intake, hence it contains no missing nutrient values, an ideal setup to train machine learning models [5]. Since 2017, the USDA has been harmonizing these different data sources in FoodData Central (FDC), labeling SR data as SR-Legacy[6]. In particular, SR28 (released in 2015) is the final version of SR-Legacy databases, and it is the foundation of FNDDS 2015-2016. In addition to FNDDS and SR-Legacy, FoodData Central stores also Foundation Foods, a new food composition dataset that reports individual sample measurements behind the nutrient average values that populate the other databases, and metadata reporting the number of samples, location, time-stamps, analytical methods used, and if available, cultivar and production practices.

As shown in Figure S1, for the years 2007-2010 the USDA developed a flavonoids database for population surveys that extended the original nutritional panel of 65 nutrients to 102. For our analysis we kept all nutrients measured in g, mg or μg, dropping "Energy", "Folate, DFE" and "Vitamin A, RAE", resulting in 99 nutrients, converted to grams (g).

We chose FNDDS 2009-2010 as data training for FoodProX, as it gave us the possibility to combine the manual labels assigned by Steele et al. in [7], with the widest panel of nutrients available for population studies. Out of 7,253 foods in FNDDS 2009-2010, 2,484 food items are assigned to a unique NOVA class, while the remaining 4,769 foods are not classified (730), or need further decomposition (4,039) into 2,946 ingredients imported from the SR24 database.

Figure S2A shows the proportion of NOVA classes in the initial dataset, labels mainly derived by following the hierarchical encoding of food items provided by FNDDS. Indeed, each food is assigned to an 8-digit code, and the first five digits represent food categories. For instance, code 13230120 is assigned to "Pudding, flavors other than chocolate, ready-to-eat, sugar free" where the first digit '1' represents "Milk and Milk Products"; the first two digits '13' represents "Milk Desserts and Sauce"; and similarly '132' represents "Puddings, Custards, and other Milk Desserts." Relying on FNDDS food categories leads to two major limitations of the current

classification system: (a) the existence of many exceptions. For example, a non ultra-processed food could belong to a category assumed to contain only ultra-processed food. Resolving these exceptions is a laborious work that requires domain knowledge; (b) limited scalability, as not all databases have a fine-tuned hierarchy of categories assigned to foods comparable to FNDDS.

Additionally, we found that some unclassified items, despite being labeled as requiring further decomposition, had only one ingredient. For instance, this is the case for the unclassified item 'Egg, whole, raw' (food code 31101010), created by linking only a single food from the SR database: 'Egg, whole, raw, fresh' (SR code 1123). Hence, we migrated 478 such unclassified single-ingredient foods to the training dataset (Figure S2B).

To further improve the training dataset, we manually classified nine foods, to extend the coverage of staple ingredients like 'Salt', or poorly represented classes like meat and fish (Table S1). In particular, the addition of 'Salt' in the training helped FoodProX to better calibrate the role of sodium in identifying ultra-processed food.

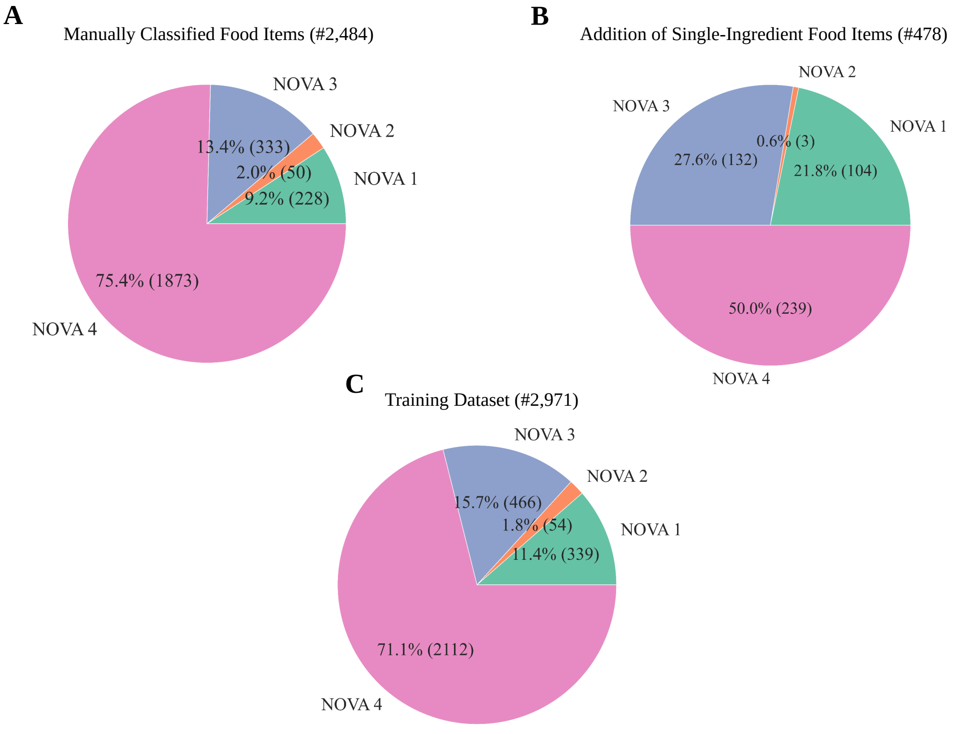

Figure S2: **Proportion of NOVA classes in the manual classification and training dataset.** (**A**) Steele et al. [7] manually assigned NOVA classes to 2,484 from 7,253 food items in FNDDS 2009-2010, (**B**) We identified 478 single-ingredient and unclassified food items in FNDDS 2009-2010 linked to a single food in the SR database with assigned NOVA labels. (**C**) Final training dataset with the corrections on single-ingredient food items and the addition of 9 manually classified food items reported in Table S1.

Table S1: Manual Additions to the Training Dataset

Food code	Food Description	Ingredients	NOVA Class
2047	Salt	• Salt, table (directly imported from SR database)	2
26100100	Fish, NS as to type, raw	• Fish, pollock, walleye, raw<br>• Fish, salmon, sockeye, raw<br>• Fish, tilapia, raw<br>• Fish, catfish, channel, farmed, raw	1
26115000	Flounder, raw	• Fish, flatfish (flounder and sole species), raw	1
26119100	Herring, raw	• Fish, herring, Atlantic, raw	1
26121100	Mackerel, raw	• Fish, mackerel, Atlantic, raw<br>• Fish, mackerel, Pacific and jack, mixed species, raw	1
26125100	Ocean perch, raw	• Fish, ocean perch, Atlantic, raw	1
26313100	Mussels, raw	• Mollusks, mussel, blue, raw	1
63123020	Grapes, American type, slip skin, raw	• Grapes, american type (slip skin), raw	1
27116400	Steak tartare (raw ground beef and egg)	• Beef, ground, 85% lean meat / 15% fat, raw<br>• Egg, yolk, raw, fresh<br>• Onions, raw<br>• Fish, anchovy, european, canned in oil, drained solids	3

1.3 NOVA Manual Classification Coverage

The coverage of NOVA classification for FNDDS 2001-2017 is presented in Figure S3. For over 55% of the databases, NOVA classification relies on having a precise ingredient decomposition of food items, information that is extremely uncommon. To note, the manual NOVA classification has been updated since the initial classification on FNDDS 2009-2010 used in [7]. For clarity, only for FNDDS 2009-2010 in Figure S3, we used the NOVA classification conducted

by Steele et al. in [7], which is the data source that we used to train FoodProX. However, close to the convergence of this manuscript, Steele and colleagues have updated the manual classification for FNDDS 2009-2010, and propagated the labels in different cohorts by matching the food codes through the years. In the updated manual classification of FNDDS 2009-2010 the percentage of food items relying on ingredient decomposition increased to 58.98% from 55.69%.

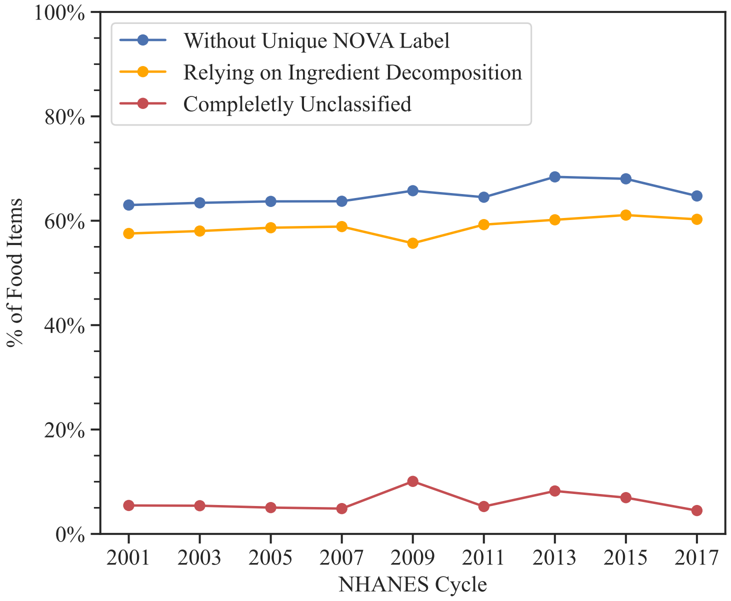

Figure S3: **Manual NOVA Classification Coverage Over FNDDS 2001-2017.** On average 35% of the food items have a manual NOVA label without relying on ingredient decomposition.

1.4 Nutrient Panels

The large nutritional panel available for FNDDS 2009-2010 allowed us to train FoodProX with varying subsets of nutrients. The widest panel consists of 99 nutrients, including the flavonoid measurements developed for NHANES 2007-2010 (Table S2) [8]. Among these 99 nutrients, we further selected and trained on 62 nutrients common to NHANES 2001-2018 (Table S3), and 58 nutrients available in NHANES 1999-2018 (Table S4). Figures 1C-1D and Figures 2A-2C in the manuscript describe the results on FNDDS 2009-2010 with 99 nutrients, while Figure 2D is related to the analysis of FNDDS 2015-2016, therefore using a nutrient panel of 62 nutrients. For the epidemiological analysis in Section S3, leveraging data from 1999 to 2006, we opted for 58 nutrients.

With the goal to tackle branded products and the consumer space, we additionally trained on a subset of 12 nutrients contributing to FDA nutrition facts (Table S5), excluding calories and total amount of trans fatty acids, as the latter is not available in the original batch of 99 nutrients.

All nutrients were log-transformed and 0 values were substituted with e^{-20}, a choice justified by the orders of magnitude spanned by nutrient concentrations in food [9].

Table S2: 99 Nutrient Panel for NHANES 2007-2010

**Nutrients**			**37 Additional Nutrients From FNDDS Flavoniod 2007-2010 Database**	
Protein	Vitamin E (alpha-tocopherol)	6:0	Total flavonoids	Eriodictyol
Total Fat	Vitamin D (D2 + D3)	8:0	Cyanidin	Hesperetin
Carbohydrate	Cryptoxanthin, beta	10:0	Petunidin	Naringenin
Alcohol	Lycopene	12:0	Delphinidin	Total flavanones
Water	Lutein + zeaxanthin	14:0	Malvidin	Apigenin
Caffeine	Vitamin C	16:0	Pelargonidin	Luteolin
Theobromine	Thiamin	18:0	Peonidin	Total flavones
Sugars, total	Riboflavin	18:1	Total anthocyanidins	Isorhamnetin
Fiber, total dietary	Niacin	18:2	(+)-Catechin	Kaempferol
Calcium	Vitamin B-6	18:3	(-)-Epigallocatechin	Myricetin
Iron	Folate, total	20:4	(-)-Epicatechin	Quercetin
Magnesium	Vitamin B-12	22:6 n-3	(-)-Epicatechin 3-gallate	Total flavonols
Phosphorus	Choline, total	16:1	(-)-Epigallocatechin 3-gallate	Daidzein
Potassium	Vitamin K (phylloquinone)	18:4	Theaflavin	Genistein
Sodium	Folic acid	20:1	Thearubigins	Glycitein
Zinc	Folate, food	20:5 n-3	Theaflavin-3,3'-digallate	Total isoflavones
Copper	Vitamin E, added	22:1	Theaflavin-3'-gallate	
Selenium	Vitamin B-12, added	22:5 n-3	Theaflavin-3-gallate	
Retinol	Cholesterol	Fatty acids, total monounsaturated	(+)-Gallocatechin	
Carotene, beta	Fatty acids, total saturated	Fatty acids, total polyunsaturated	Total catechins (monomeric flavan-3-ols only)	
Carotene, alpha	4:0		Total flavan-3-ols	

Table S3: 62 Nutrient Panel for NHANES 2001-2018 Cycles

**Nutrients**		
Protein	Vitamin E (alpha-tocopherol)	6:0
Total Fat	Vitamin D (D2 + D3)	8:0
Carbohydrate	Cryptoxanthin, beta	10:0
Alcohol	Lycopene	12:0
Water	Lutein + zeaxanthin	14:0
Caffeine	Vitamin C	16:0
Theobromine	Thiamin	18:0
Sugars, total	Riboflavin	18:1
Fiber, total dietary	Niacin	18:2
Calcium	Vitamin B-6	18:3
Iron	Folate, total	20:4
Magnesium	Vitamin B-12	22:6 n-3
Phosphorus	Choline, total	16:1
Potassium	Vitamin K (phylloquinone)	18:4
Sodium	Folic acid	20:1
Zinc	Folate, food	20:5 n-3
Copper	Vitamin E, added	22:1
Selenium	Vitamin B-12, added	22:5 n-3
Retinol	Cholesterol	Fatty acids, total monounsaturated
Carotene, beta	Fatty acids, total saturated	Fatty acids, total polyunsaturated
Carotene, alpha	4:0	

Table S4: 58 Nutrient Panel for NHANES 1999-2018 Cycles

Nutrients		
Protein	Vitamin E (alpha-tocopherol)	14:0
Total Fat	Cryptoxanthin, beta	16:0
Carbohydrate	Lycopene	18:0
Alcohol	Lutein + zeaxanthin	18:1
Water	Vitamin C	18:2
Caffeine	Thiamin	18:3
Theobromine	Riboflavin	20:4
Sugars, total	Niacin	22:6 n-3
Fiber, total dietary	Vitamin B-6	16:1
Calcium	Folate, total	18:4
Iron	Vitamin B-12	20:1
Magnesium	Vitamin K (phylloquinone)	20:5 n-3
Phosphorus	Folic acid	22:1
Potassium	Folate, food	22:5 n-3
Sodium	Cholesterol	Fatty acids, total monounsaturated
Zinc	Fatty acids, total saturated	Fatty acids, total polyunsaturated
Copper	4:0	
Selenium	6:0	
Retinol	8:0	
Carotene, beta	10:0	
Carotene, alpha	12:0	

Table S5: 12 Nutrient Panel for Branded Products

Nutrients
Protein
Total Fat
Carbohydrate
Sugars, total
Fiber, total dietary
Calcium
Iron
Sodium
Vitamin C
Cholesterol
Fatty acids, total saturated
Total Vitamin A = Retinol + Carotene, beta + Carotene, alpha + Cryptoxanthin, beta

1.5 Hierarchical Clustering of Foods according to Nutrients

Leveraging the 99 nutrient panel for FNDDS 2009-2010, we clustered all foods in an unsupervised fashion, using the dynamic tree cut algorithm [10]. The algorithm retrieves 20 clusters, annotated in the first column of the cluster map shown in Figure S4. The nutrient-derived clusters are not good predictors of NOVA classes, both computationally-derived (Column 2) and manually-assigned (Column 3). We measure the mutual dependence between different clustering methodologies with adjusted mutual information (AMI), an adjustment of the classic mutual information (MI) to account for chance, that takes a value of 1 when two partitions are identical and 0 when the MI between two partitions equals the value expected by chance [11]. In particular, for the foods in the training data which were manually-assigned to NOVA 1, 2, 3, and 4,

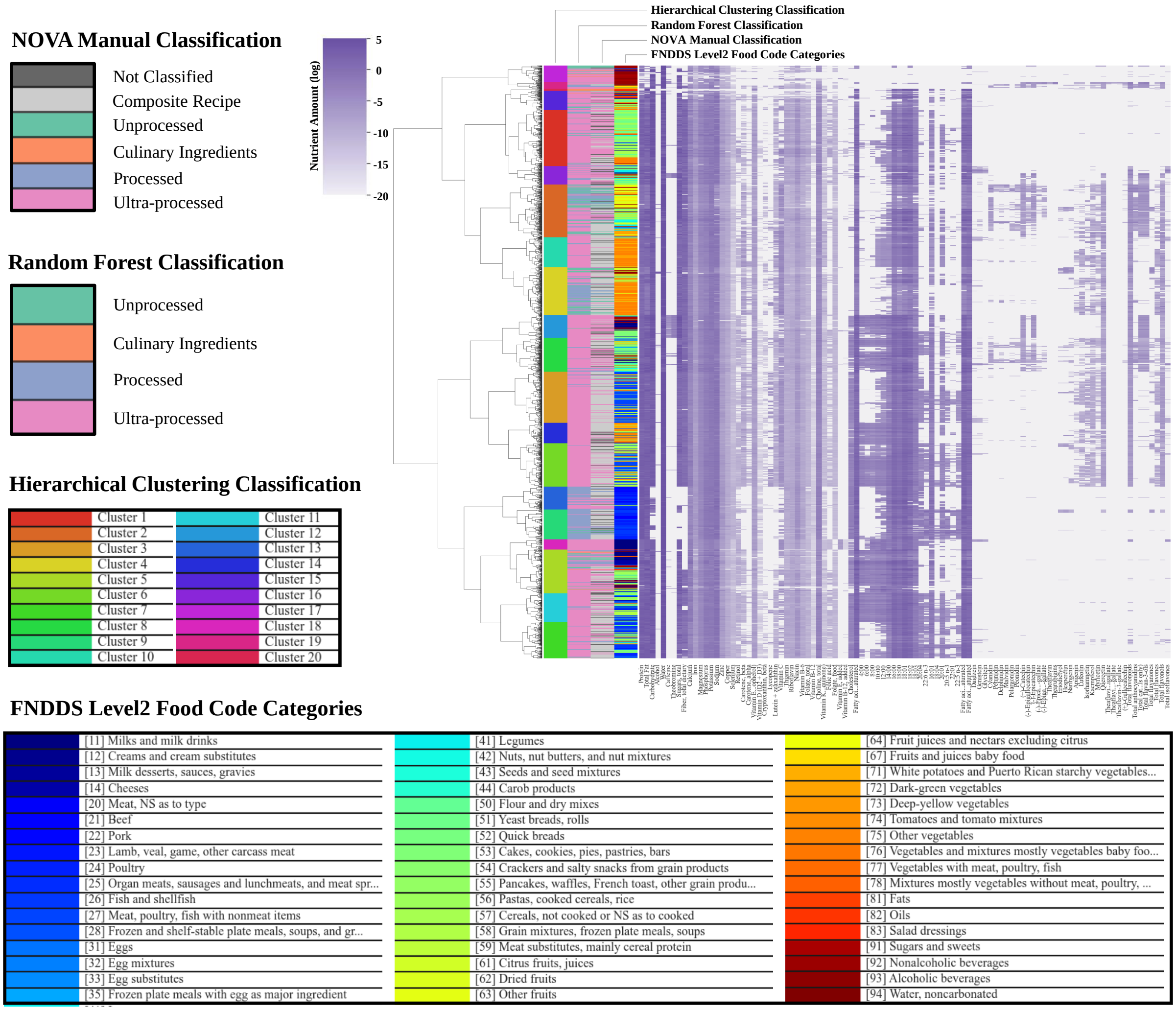

Figure S4: **Hierarchical clustering of foods according to nutrient content.** We clustered all foods in FNDDS 2009-2010, each represented by a vector of 99 log-transformed nutrients. The cluster map is annotated according to different classification strategies. In Column 1, starting from the left, each color corresponds to a cluster found by the dynamic tree-cut algorithm. In Column 2 we report the predicted NOVA classes by FoodProX, while Column 3 encodes the manual labels used during the training phase of FoodProX. Finally, in Column 4 items are color-coded according to the first two digits of their FNDDS food codes.

we find AMI=0.12. Similarly, for the classes predicted by FoodProX we obtain AMI=0.14. The nutrient-based hierarchical clustering is more consistent with the first two digits of the FNDDS food codes (Column 4), capturing broad food groups as defined by the database (AMI=0.39). This result suggests that the performance of FoodProX is not merely induced by the classification of foods based on nutrient content, but FoodProX combines in a non-linear fashion the features of processing techniques (supervised information learned from NOVA manual labels), with food composition data (unsupervised information learned from FNDDS nutritional values). Similar results were obtained with 62, 58, and 12 nutrient panels described in Section S1.4.

2 Random Forest Classifier FoodProX and Food Processing Score $FPro$

2.1 Random Forest Classifier

FoodProX is based on a Random Forest Classifier whose hyper-parameters were chosen using the python *sklearn RandomizedSearchCV* function with a 5-fold stratified cross-validation. In particular, the sampled search considered a number of trees between 200 and 2000, a maximum number of features equal to $\surd$ or to $\log_2$, and a maximum depth of the trees between 100 and 500 (tested with 99 nutrients). Over 100 random samples, the function picked a number of estimators equal to 200, a maximum number of features equal to $\surd$, and a maximum tree depth equal to 420 (currently used). Further runs of the random search found other combinations of parameters spanning the whole search intervals, suggesting an overall robust performance of the classifier, independently from the hyper-parameter tuning.

We evaluated the performance and stability of FoodProX over a 5-fold stratified cross-validation of the labeled dataset (Figure S13C), with varying input resolution. In Figures S13A-H we show the ROC curves and Precision-Recall curves for each NOVA class, while in Tables S6A and S6B we report the average and standard deviation of AUC and AUP over the 5 folds, and across the different nutrient panels, as reported in the manuscript. The high performance for different nutrient resolutions is encouraging, as for many foods we lack access to an extensive panel of nutrients.

To improve the performance of the classifier on new data and limit over-fitting, we retrained it using SMOTE [12] to correct for the unbalance in class representation, and created an ensemble voting system of 5 classifiers trained on 4/5 of the generated data. The predictions on unseen data are then calculated as the average of the 5 classifiers.

Table S6: **AUC and AUP for the four NOVA classes**. For each NOVA class, we report the average and standard deviation of AUC and AUP over the stratified 5-folds, for 12, 62, and 99 input nutrients. We summarize the results across nutrient panels of different resolutions in bold.

(A)

	NOVA 1	NOVA 2	NOVA 3	NOVA 4
Average AUC Nutrition Facts	0.981662	0.966348	0.967094	0.976772
Std AUC Nutrition Facts	0.003621	0.044898	0.010411	0.003432
Average AUC 62 Nutrients	0.980837	0.962878	0.970605	0.979866
Std AUC 62 Nutrients	0.00173	0.048586	0.00806	0.004876
Average AUC 99 Nutrients	0.978806	0.960406	0.971156	0.980085
Std AUC 99 Nutrients	0.002772	0.052151	0.007394	0.004237
**Average AUC**	0.980435	0.963211	0.969618	0.978908
**Std AUC**	0.0012	0.002437	0.001799	0.001513

(B)

	NOVA 1	NOVA 2	NOVA 3	NOVA 4
Average AUP Nutrition Facts	0.891112	0.756997	0.864605	0.990558
Std AUP Nutrition Facts	0.035105	0.169225	0.040566	0.001414
Average AUP 62 Nutrients	0.891971	0.736245	0.873025	0.991702
Std AUP 62 Nutrients	0.024412	0.185744	0.03922	0.002178
Average AUP 99 Nutrients	0.881419	0.74707	0.879123	0.991783
Std AUP 99 Nutrients	0.024092	0.193387	0.032601	0.001954
**Average AUP**	0.888168	0.74677	0.872251	0.991348
**Std AUP**	0.004785	0.008475	0.005952	0.00056

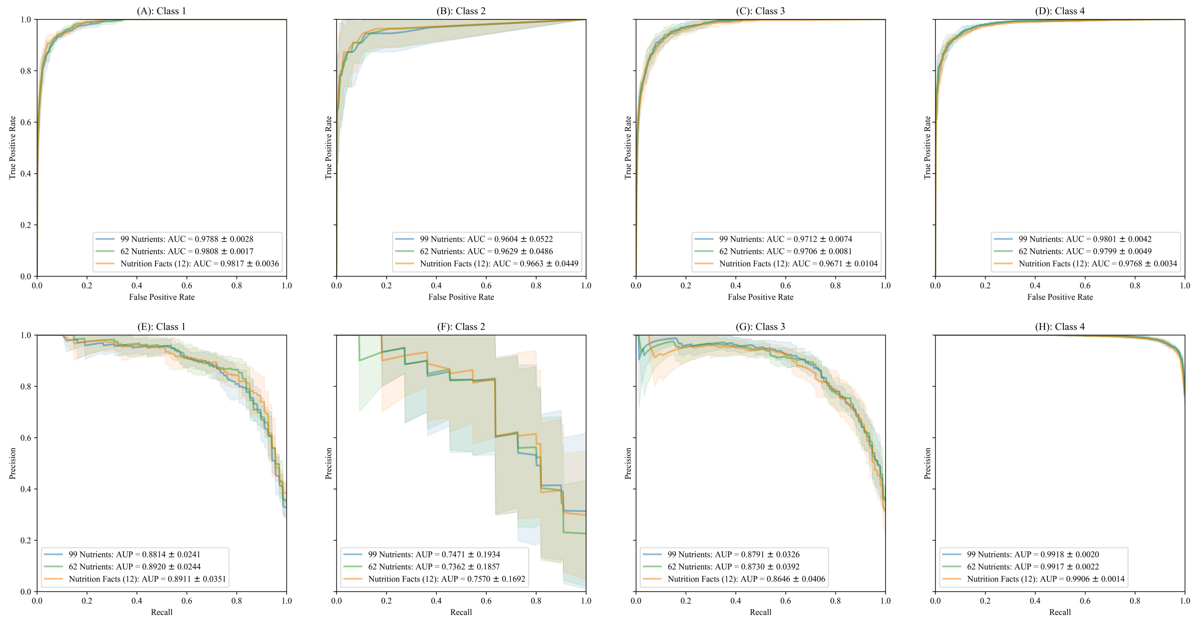

Figure S5: **Random forest performance over the 4 NOVA classes**. For each NOVA class, we evaluated the performance of the random forest classifier with a 5-fold cross-validation. We observe similar performances for the classifiers trained with 99, 62, and 12 nutrients. In Panels A-D we show the ROC curves for each NOVA class, while panels E-F display the Precision-Recall curves.

2.2 Feature Importance

Inspired by the work of Parr et al. [13], we investigated how different nutrients contribute to the overall performance of FoodProX, i.e., their feature importance. The most popular way to assess feature importance in the random forest algorithm is the mean decrease impurity, measuring how effective a feature is at reducing uncertainty (classifiers) or variance (regressors) when building the decision trees. However, this methodology is not reliable when potential predictors vary in their scale of measurement or their number of categories [14]. We opted for permutation feature importance, a technique quantifying the relevance of each feature by permuting the specific input column and measuring the decrease in accuracy or R^2 compared to the baseline. This approach handles also the presence of collinear features, i.e., variables with some significant degree of linear or nonlinear dependence, that should be clustered and permuted together.

First, we modified the algorithm to work on a stratified 5-fold cross-validation, with data splits consistent with the cross-validation for the baseline model. Second, we addressed the high degree of collinearity of the nutrient space by removing all measurements like "Total Fat" or "Total flavonoids", as they represent straightforward linear combinations of other nutrients. On the reduced set of 85 nutrients, we studied both rank correlation and feature dependence, i.e., the extent to which each feature can be predicted by the others through a random forest regressor. We cluster together features with $\rho_{Spearman} \geq 0.80$, and additionally, every nutrient well fitted

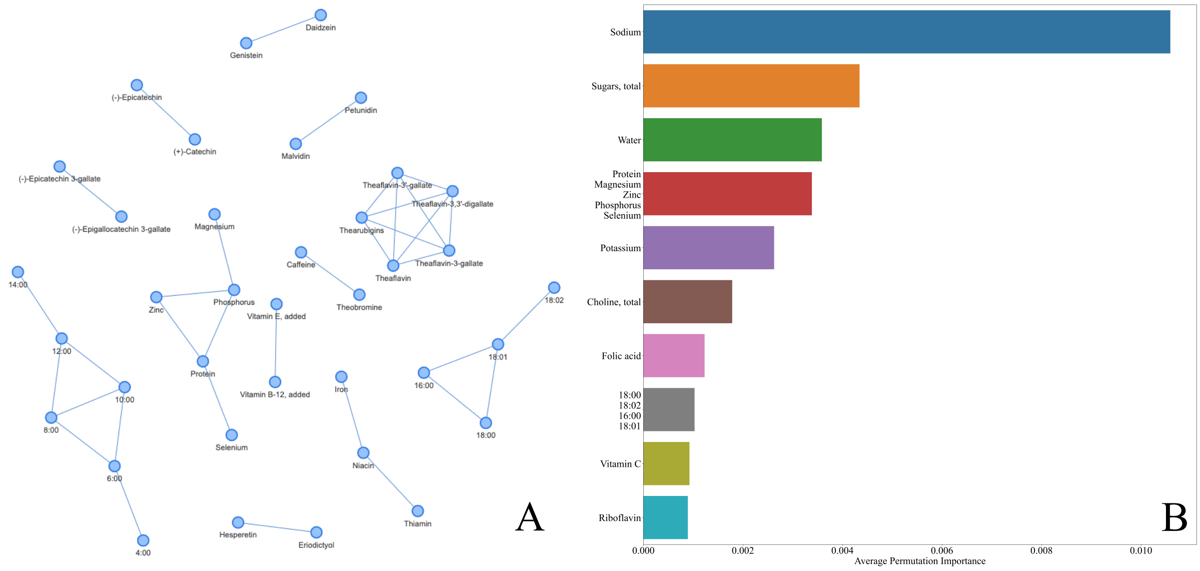

Figure S6: **Permutation feature importance**. (a) Clusters of features with strong dependence. We cluster together features with $\rho_{Spearman} \geq 0.80$, and additionally, every nutrient well predicted by the remaining nutrient set through a random forest regressor is connected to each of the independent variables determining a drop ≥ 0.80 in the coefficient of determination. Each connected component determines a single cluster of features permuted at the same time, while isolated nutrients (not shown) are permuted on their own. (b) Top 10 most important nutrient clusters sorted by average permutation importance over 20 reshuffles.

by the random forest regressor is connected to each of the nutrients in the independent variable set that determines a drop ≥ 0.80 in the coefficient of determination (Figure S6A). Given the stochastic nature of the permutation feature importance, we repeated the estimation 20 times and ranked the feature clusters according to their average drop in accuracy. In Figure S6B we report the top 10 most significant feature clusters, where only Sodium shows more predictive power than the other nutrients, suggesting that there is no single nutrient marker for food processing.

We further investigated the role of each nutrient i in determining the prediction for a selected food f, by using SHAP [15], over 5-fold stratified cross-validation. The SHAP explanation method computes Shapley values from coalition game theory. The feature values of a data instance act as players in a coalition, and Shapley values indicate how to fairly distribute the "payout", i.e., the prediction, among the features. For each food f and NOVA class c, SHAP specifies the explanation as

$$p_c(f) = \langle p_c(f) \rangle + \sum_j SHAP\ value_j^c(f). \tag{1}$$

A player in the game could be also a group of features, as we previously investigated for the permutation feature importance analysis. However, due to high computational complexity, we focused on single nutrients, despite the presence of collinear features. In Figure S7 we show the top 10 nutrients in terms of $\langle |SHAP\ value_j^c(f)| \rangle$ for each NOVA class c. The magnitude

of the explanations varies significantly across NOVA classes, with only Sodium and Folic acid exhibiting distinct behaviors. For instance, when the amount of Folic acid is high, p_4 is expected to be high as well, while the other NOVA classes display lower probabilities. In the case of Sodium, high values are likely to increase p_4 and decrease p_1 and p_2, while the processed class NOVA 3 displays mixed behaviors. Overall, we find that the ranking of SHAP values is in good overlap with Figure S6B.

To understand the statistical relevance of SHAP explanations, we introduced a new positive variable

$$relevance_c^i(f) = \frac{|SHAP\ value_i^c(f)|}{\sum_j |SHAP\ value_j^c(f)|}, \tag{2}$$

measuring to which extent nutrient i contributes to the superior limit of the absolute difference $|p_c(f) - \langle p_c(f) \rangle|$. In Figure S8, for each NOVA class c we display the violin-plot of $relevance_c^i$ for the top 10 nutrients in terms of median effect. Each violin-plot is compared with the quantiles of a maximum entropy null model for feature relevance, i.e., a Dirichlet multivariate distribution with marginal probability for each feature equal to a beta distribution with parameters $\alpha = 1$ and $\beta = n_{features} - 1$[16]. The higher the overlap between a violin plot and the null model ranges, the closer we are to a scenario lacking driving nutrients in determining the final class probability. In Figure S8, we observe a huge variability in the feature relevance of each nutrient, as captured by the shape of the violin plots, evidence of the lack of strong driving signals in the model decision-making. As expected, the largest nutrient contributions are found for NOVA 1 and NOVA 4, the two extreme processing classes.

2.3 Food Processing Score $FPro$

The classifier probability space is a 4-D probability simplex that collects all vectors satisfying

$$\{\overrightarrow{p} \in \mathbb{R}^4,\ p_1 + p_2 + p_3 + p_4 = 1, \quad p_i \geq 0\ \forall i\}. \tag{3}$$

We define the processing score $FPro_k$ as the projection of any food $\vec{p}_k = (p_1^k, p_2^k, p_3^k, p_4^k)$ over the line going from the pure minimally-processed state $\overrightarrow{p}_{MP} = (1,0,0,0)$ to the pure ultra-processed state $\overrightarrow{p}_{UP} = (0,0,0,1)$, represented by the parametric equation

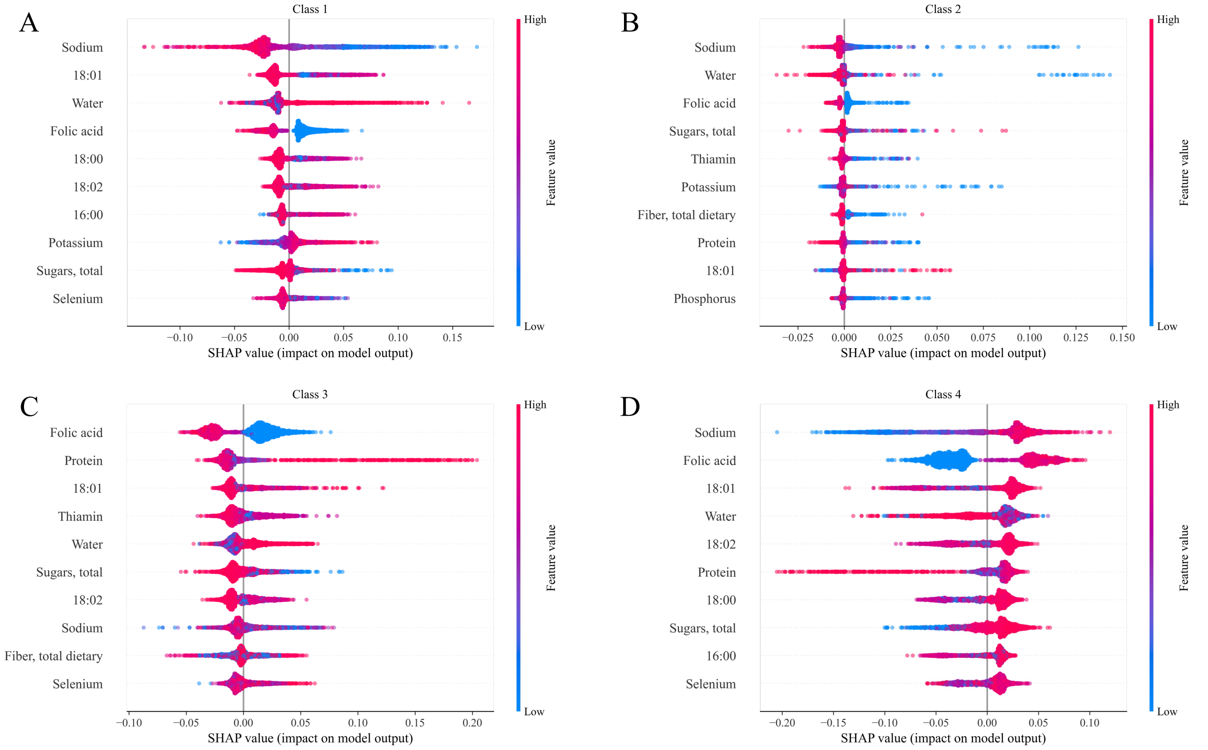

Figure S7: **Shapley values**. Top 10 nutrients in terms of average $\langle |SHAP\ value_j^c(f)| \rangle$ over all foods f, for class (a) NOVA 1, (b) NOVA 2, (c) NOVA 3, and (d) NOVA 4 (see Eq. S1). For each NOVA class c, the color scale correlates with the class probability p_c. The number of data points in the beeswarm plots is equivalent to the size of the training set represented in Figure S2C (2,971).

$$\overrightarrow{l(t)} = \begin{bmatrix} 1 \\ 0 \\ 0 \\ 0 \end{bmatrix} + t \begin{bmatrix} -1 \\ 0 \\ 0 \\ 1 \end{bmatrix}, \tag{4}$$

equivalent to the explicit equation $p_1 = 1 - p_4$. The orthogonal projection of food $\overrightarrow{p}_k$ follows as the intersection between Eq. S4 and the plane passing through $\overrightarrow{p}_k$ and orthogonal to $\overrightarrow{l(t)}$, i.e.,

$$-p_1 + p_4 + p_1^k - p_4^k = 0. \tag{5}$$

The parameter t^* satisfying Eqs. S4-S5 determines the processing score $FPro_k$ in Eq. 1. Of note, $FPro$ assigns a value around 0.5 for all NOVA 2/3 classes, which are then differentiated using the p_2/p_3 ratio.

We focused on the extreme classes NOVA 1 and NOVA 4, as by definition [17], they are the only ones with a clear "natural" ranking, ideal to define a processing scale. Indeed, NOVA 3 is not more processed than NOVA 2, they simply collect remarkably different items, according

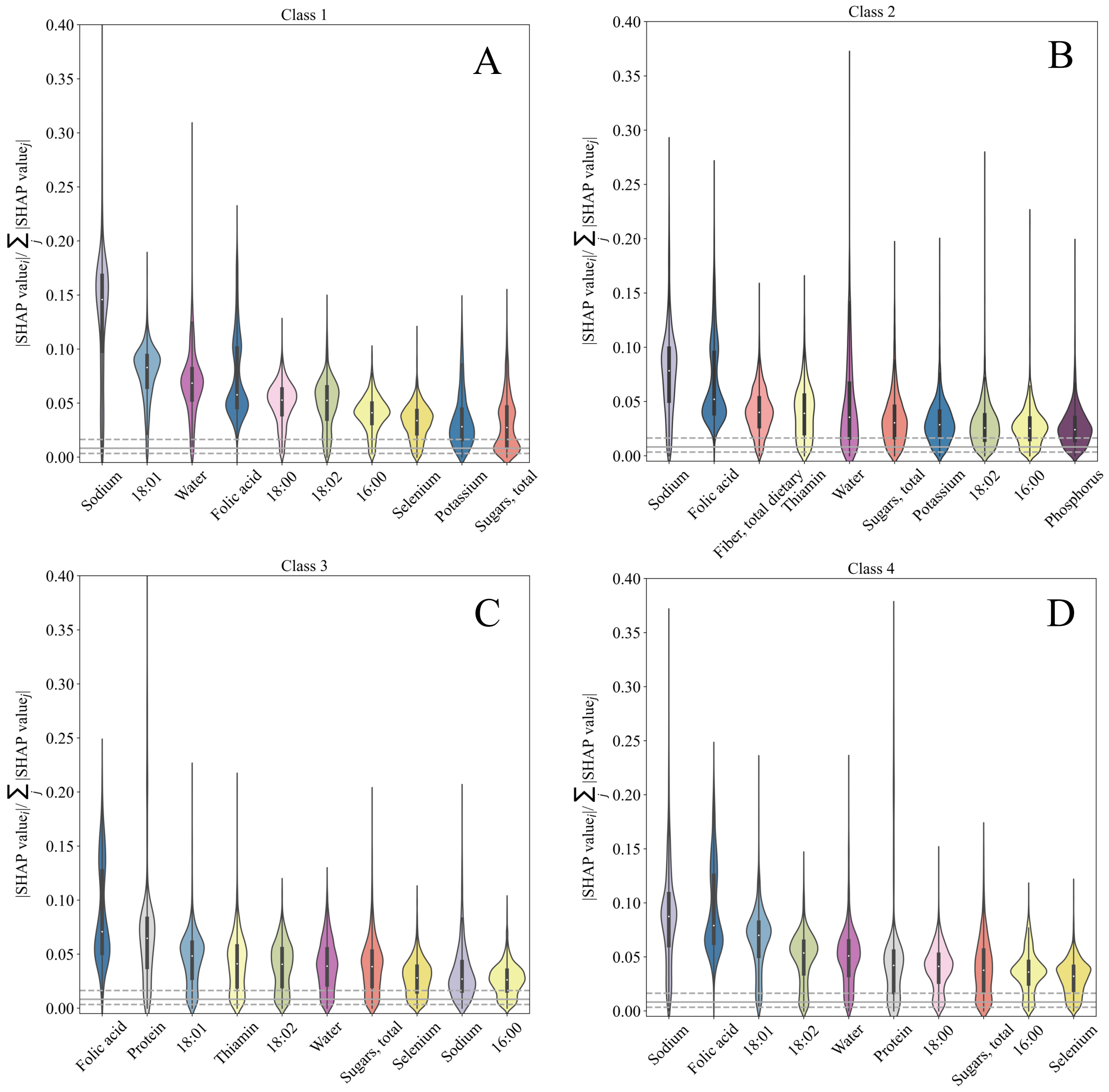

Figure S8: **Relevance of Shapley values**. Top 10 nutrients in terms of median $relevance_c^i(f)$ across all foods f, for class (a) NOVA 1, (b) NOVA 2, (c) NOVA 3, and (d) NOVA 4 (see Eq. S2). The gray dashed lines correspond to the quantiles Q_1 and Q_3 of a beta distribution with parameters $\alpha = 1$ and $\beta = n_{features} - 1$. The full grey line points to the median of the same null model. The number of data points captured by the violin plots is equivalent to the size of the training set represented in Figure S2C (2,971). In each violin plot, the white point represents the median, the tick gray bar captures the interquartile range (upper quartile Q_3 - lower quartile Q_1), and the thin gray line represents the remaining part of the distribution.

to nutrient composition and consumed portion. However, both classes are more complex than NOVA 1, and less processed than NOVA 4. While it is true that a "pure" NOVA 2/3 item would have $FPro = 0.5$, in real-world data, this is an uncommon scenario, so the extent of the residual probabilities p_1 and p_4 measures if a product is leaning towards the minimally processed or the ultra-processed extreme.

2.4 Validation of *FPro* in Different FNDDS Editions

We investigated the relation of *FPro*, trained on FNDDS 2009-2010 with a 62 nutrient panel, and NOVA manual classification in other editions of FNDDS, to control for any potential overfitting and validate the performance of our algorithm on new foods, or foods whose nutrient content has changed over time. In particular, in Figure S9 we show the results for FNDDS 2015-2016, so far, the USDA database for dietary studies with the highest number of food items. All four classes of manually labeled items correspond to well-localized and distinguishable distributions of *FPro*.

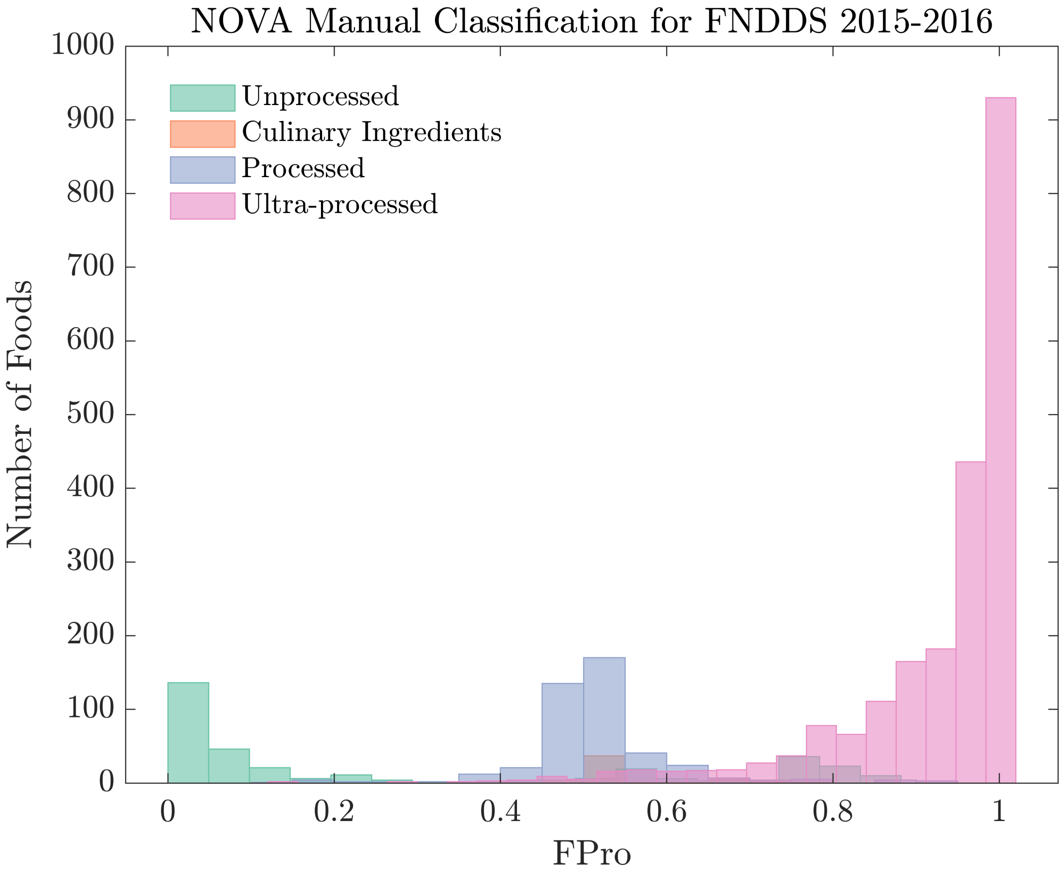

Figure S9: **Food processing score for NOVA manual labels in 2015-2016**. Variability of *FPro* (trained on FNDDS 2009-2010) within manual NOVA classes for FNDDS 2015-2016.

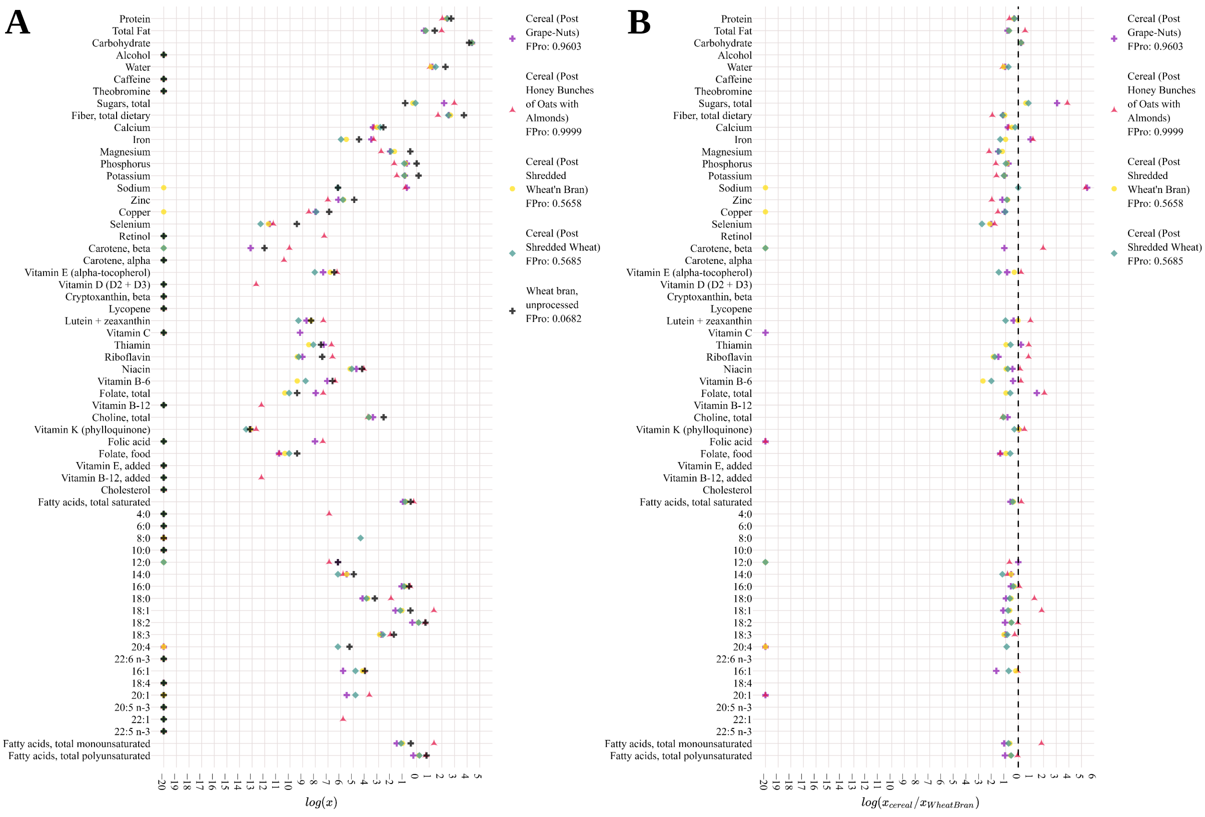

Figure S10: **Nutrient profiles of Post cereals compared to "Wheat bran, unprocessed"**. (a) All 62 nutrients measured in FNDDS 2015-2016 are shown in log-scale, and with varying colors and markers depending on the food item. (b) We rescale the nutrient profile of each cereal by the corresponding value per 100 grams found in unprocessed wheat bran and plot them in log-scale. The black dashed line corresponds to 1, i.e., identical nutrient content.

2.5 Case Study on Post Cereals.

To further investigate the interplay between nutrient patterns and $FPro$, we collected the 62 nutrient profiles describing each Post cereal highlighted in Figure 2, and compared them with the nutritional values for 100 grams of unprocessed wheat bran ($FPro$=0.0682). We chose this specific ingredient as the Post Shredded Wheat 'N Bran contains whole grain wheat, wheat bran, and the antioxidant Butylated hydroxytoluene [18], and we tried to find any potential matching item in FNDDS 2015-2016. In Figure S10, we compare the nutrient values in absolute terms (Panel A), or as the ratio with their counterpart in wheat bran (Panel B). Interestingly, we observe how the pattern of alterations involves all nutrients, increasing the level of $FPro$ even for simple products like Post Shredded Wheat 'N Bran, as its nutrient profile is not characteristic of any natural ingredient per 100 grams, but it corresponds to a mildly processed food ($FPro$=0.5658).

2.6 Source of Food

The location where a food was prepared, as well as the origin of the ingredients, could be indicative of its degree of processing. To investigate this hypothesis we used the variable

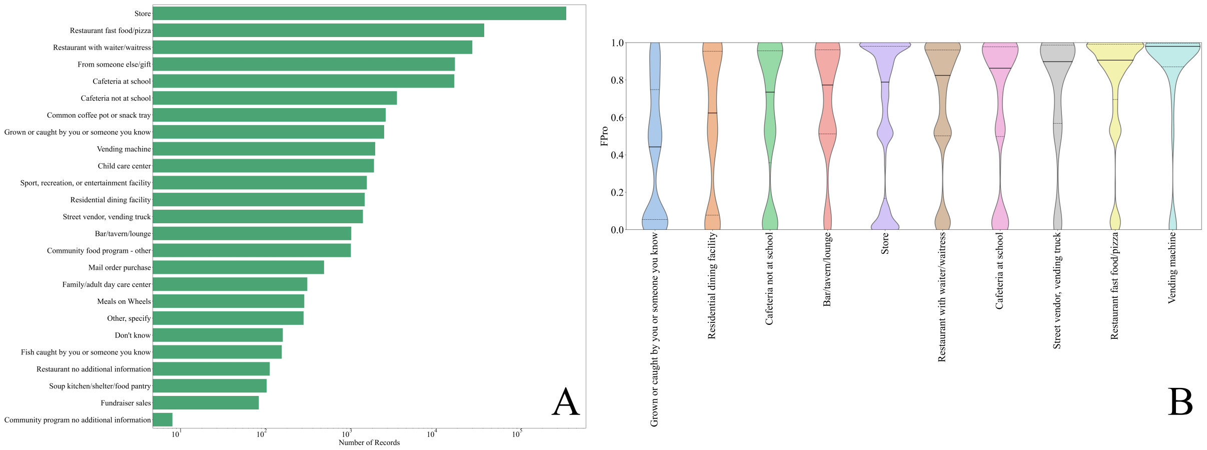

Figure S11: ***FPro* stratified by food source in NHANES 2003-2005.** (a) Food sources reported in the cohorts, sorted in descending order by number of records. (b) Violin plots of 10 selected food sources ranked in increasing order of median *FPro*. Sample sizes are consistent with the number of records shown in panel (a), with a minimum of 1,132 data points for "Bar/tavern/lounge" and a maximum of 378,420 data points for "Store". We annotate median values with full lines, and lower quartiles and upper quartiles with dashed lines. In each violin plot, the bold line represents the median, and the dashed lines represent the lower quartile Q_1 and the upper quartile Q_3. Source data are provided in Source Data Supplementary Figure 11a and 11b.xlsx.

DR1FS in NHANES, corresponding to the question "Where did you get (this/most of the ingredients for this)?", and available for the years 2003-2018 (`https://wwwn.cdc.gov/Nchs/Nhanes/2005-2006/DR1IFF_D.htm#DR1FS`). By leveraging our analysis of the merged NHANES cohorts between 1999 and 2006 (Section S3.2), we were able to stratify *FPro* by food source for two cycles (2003-2004, 2005-2005). In Figure S11A we report the 25 different types of food sources found in the population, sorted by decreasing number of records. As expected, "Store" contributes to the majority of the records, followed by "Restaurant fast food/pizza". In Figure S11B we selected 10 of the most popular food sources, and visualize the related distributions of *FPro* in increasing order of median. Overall, *FPro* is significantly different across source categories, as quantified by the Kruskal-Wallis H-test (p-value $< 10^{-15}$), suggesting that the overlap between the source categories (driven by foods with multiple origins), while present, is limited. We also compared each pair of source categories with the Mann-Whitney U rank test, finding that (Cafeteria not at school, Bar/tavern/lounge), (Cafeteria at school, Bar/tavern/lounge), (Store, Bar/tavern/lounge), and (Restaurant with waiter/waitress, Bar/tavern/lounge) do not survive multiple testing with Bonferroni correction ($\alpha = 0.01$), indicating continuous distributions with equal medians. Additionally, to control for "overpowering", i.e., the scenario in which large samples almost surely determine a statistically significant outcome, we estimated the effect size r following [19]. Across the 45 combinations of food sources, 23 show $r \geq 0.1$ (*small* effect), 6 have $r \geq 0.3$ (*medium* effect), and the pair ("Grown or caught by you or someone you know", "Vending machine") is characterized by the largest effect size with $r = 0.5798$.

3 Individual Diet Processing Scores $iFPro$ and Exposome

3.1 Individual Processing Score $iFPro$

To capture the extent of processing in individuals' diets we focus on two weighting schemes: a calorie-based score (Eq. 2), and a gram-based score,

$$iFPro^j_{WG} = \sum_k^{D_j} \frac{w^j_k}{W^j} FPro_k, \tag{6}$$

where D_j is the number of dishes consumed by individual j, W^j is her total amount of food in grams, and w^j_k is the amount of grams consumed for each food item (excluding water consumption, see Section S3.4).

3.2 Population Characteristics

NHANES captures a variety of information ranging from demographics and dietary intake, to lab and physical examinations. This wealth of information is compiled into hundreds of publicly available data files, that all together provide over 1,000 variables.

To investigate the relation between $iFPro$ and health, we focused on NHANES 1999-2006 exposome and phenome database, a harmonized dataset created by Patel et al. in [20], merging 255 data files from four cycles of NHANES, for a total of 41,474 individuals and 1,191 variables. The summary statistics for $iFPro_{WC}$ and $iFPro_{WG}$, characterizing the 20,047 adults (18+) in the cohort, are presented in Tables S7 and S8. All predictions are calculated with the 58 nutrient panel in Table S4.

NHANES follows the well-established two-step 24HRs dietary recall interviews to sample the dietary intake of the American population [21, 22]. The first step is done in person with a dietitian interviewing each applicant, while ensuring the highest quality of dietary recall over the past 24HRs. The second step consists of a phone interview within 3-10 days to capture a second dietary recall [23]. The multiple 24HRs dietary recalls have proved to be an effective method in the assessment of trends over the dietary intakes of individuals [24]. For all individuals who completed two-day dietary recalls (in-person and phone interview) we calculated a daily average $iFPro$, while for the remaining participants we used just the data from the in-person interview. The relevance of each individual for population statistics is based on survey weights [25, 26].

Table S7: **Population characteristics for $iFPro_{WC}$**

	mean	$iFPro_{WC}$				
		0.0 – 0.2	0.2 – 0.4	0.4 – 0.6	0.6 – 0.8	0.8 – 1.0
Counts Subjects (n = 20,047)		4	63	1487	11180	7313
Age — mean±SE	45.28±0.3	58.01±6.16	47.2±3.06	51.4±0.64	47.36±0.37	41.3±0.3
Poverty Income Ratio — mean±SE	2.99±0.04	2.58±0.98	2.92±0.3	2.97±0.07	3.06±0.04	2.88±0.05
Calories Consumed — mean±SE	2189.13±10.36	534.25±162.24	1359.03±184.71	1772.85±29.81	2143.11±13.16	2329.7±15.07
BMI — mean±SE	28.13±0.1	26.48±1.63	29.33±1.33	27.45±0.28	27.92±0.1	28.53±0.13
female — count (%)		4 (1)	41 (0.65)	879 (0.59)	5798 (0.52)	3763 (0.51)
white — count (%)		0 (0)	20 (0.32)	663 (0.45)	5389 (0.48)	3525 (0.48)
black — count (%)		0 (0)	21 (0.33)	227 (0.15)	2160 (0.19)	1810 (0.25)
mexican — count (%)		2 (0.5)	15 (0.24)	443 (0.3)	2737 (0.24)	1460 (0.2)
other hispanic — count (%)		0 (0)	6 (0.1)	86 (0.06)	467 (0.04)	270 (0.04)

Table S8: **Population characteristics for $iFPro_{WG}$**

	mean	$iFPro_{WG}$				
		0.0 – 0.2	0.2 – 0.4	0.4 – 0.6	0.6 – 0.8	0.8 – 1.0
Counts Subjects (n = 20,047)		182	2699	7286	7230	2650
Age — mean±SE	45.28±0.3	52.81±1.55	54.78±0.61	49.11±0.38	41.5±0.34	34.71±0.3
Poverty Income Ratio — mean±SE	2.99±0.04	3.01±0.15	3.13±0.05	3.15±0.05	2.92±0.04	2.56±0.07
Calories Consumed — mean±SE	2189.13±10.36	1490.34±70.36	1821.13±19.67	2134.69±14.34	2342.11±17.73	2356.4±30.02
BMI — mean±SE	28.13±0.1	28.08±0.82	27.47±0.15	27.76±0.13	28.37±0.1	29.14±0.19
female — count (%)		112 (0.62)	1618 (0.6)	3851 (0.53)	3594 (0.5)	1310 (0.49)
white — count (%)		107 (0.59)	1682 (0.62)	3790 (0.52)	2921 (0.4)	1097 (0.41)
black — count (%)		20 (0.11)	294 (0.11)	1189 (0.16)	1860 (0.26)	855 (0.32)
mexican — count (%)		37 (0.2)	529 (0.2)	1731 (0.24)	1873 (0.26)	487 (0.18)
other hispanic — count (%)		9 (0.05)	100 (0.04)	295 (0.04)	320 (0.04)	105 (0.04)

3.3 Correlation between $iFPro_{WG}$, $iFPro_{WC}$, and HEI-15

While we find an overall agreement in the population ranking determined by $iFPro_{WG}$ and $iFPro_{WC}$ ($\rho_{Spearman} = 0.7029$, Figure S12A), the two measures show significantly different patterns in epidemiological associations, in particular regarding chemical exposures (Figure S20). Indeed, a weight-based index could capture complex dietary patterns arising from the consumption of highly processed beverages such as zero-calorie soft drinks, or any type of food contaminant whose amount is independent of the provided calories.

We compared $iFPro_{WG}$ and $iFPro_{WC}$ with the HEI-2015, a score measuring the alignment of an individual's diet with the national dietary guidelines, ranging from 0 (no alignment) to 100 (complete alignment) [27]. We followed the National Cancer Institute (NCI) to calculate HEI-15 for NHANES participants [28]. As expected, we observe negative correlations emerging (Figures S12B and C), with both $iFPro_{WG}$ ($\rho_{Spearman} = -0.4862$), and $iFPro_{WC}$ ($\rho_{Spearman} =$

−0.5575).

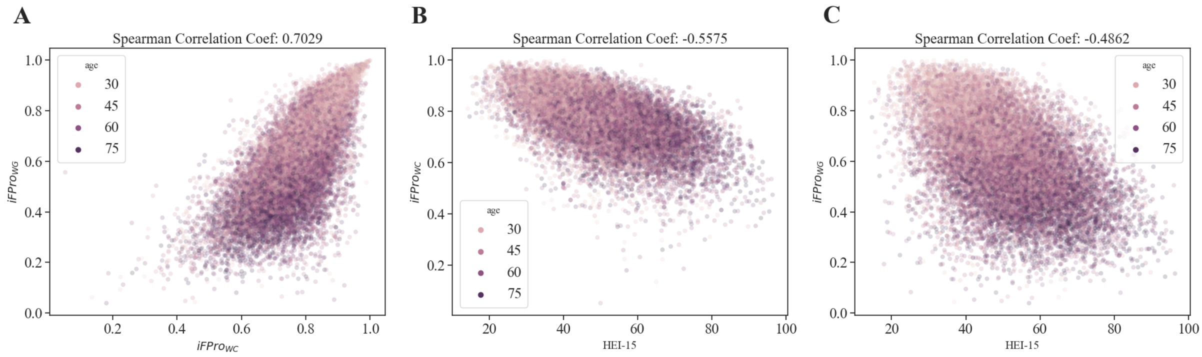

Figure S12: **Relation between $iFPro_{WC}$, $iFPro_{WG}$ and Healthy Eating Index 2015 HEI-2015**. For each individual in NHANES 1999-2006 18+ years old, we investigate the relation between (**a**) $iFPro_{WC}$ and $iFPro_{WG}$, (**b**) HEI-2015 and $iFPro_{WC}$, and (**c**) HEI-2015 and $iFPro_{WG}$, stratified by age.

Individual $\mathcal{A}$ 47 age

Day 1

**Food Code**		**Calories (kcal)**	**FPro**	**Grams**
63107010	Banana (raw)	121	0.00	136
72201100	Broccoli (raw)	30	0.02	88
75115000	Mushrooms (raw)	30	0.17	138
74101000	Tomatoes (raw)	71	0.07	394
75114000	Mixed greens salad	31	0.09	182
24124120	Fried Chicken breast	232	0.56	120
22101220	Fried Pork chop (lean)	189	0.63	117
56205008	Rice (cooked white)	663	0.69	513
61210250	Orange juice	211	0.00	450
92410550	Soft drink (caffeine add)	68	1.00	185
92302000	Tea (unsweetened)	22	0.05	2131

Day 2

**Food Code**		**Calories (kcal)**	**FPro**	**Grams**
63107010	Banana (raw)	242	0.00	272
72201100	Broccoli (raw)	17	0.00	50
75115000	Mushrooms(raw)	18	0.17	79
74101000	Tomatoes (raw)	18	0.07	101
75113000	Lettuce (raw)	8	0.02	55
42114130	Pistachio nuts (roasted , salt added)	82	0.53	15
14109010	Swiss Cheese	9	0.50	2
83106000	Italian dressing	141	0.91	59
56205008	Rice (cooked white)	268	0.69	207
28355480	Seafood soup with vegetables	185	0.69	442
41420300	Soy sauce	17	0.54	32
26158010	Tilapia (baked or broiled)	204	0.50	136
61210250	Orange juice	845	0.00	1798
93101000	Beer	310	0.51	720

Individual $\mathcal{B}$ 48 age

Day 1

**Food Code**		**Calories (kcal)**	**FPro**	**Grams**
63109010	Cantaloupe (raw)	27	0.02	78
14010100	Cheddar cheese	32	0.51	9
63223020	Strawberries (raw)	9	0.00	28
92111010	Coffee (decaffeinated)	0	0.02	192
92101000	Coffee (regular)	7	0.00	607
57230000	Cereal (Grape-Nuts)	139	1.00	39
53242000	Cookie (sugar wafer)	141	0.99	28
54337000	Cracker	80	0.99	19
25210110	Hot dog (frankfurter)	184	0.99	57
27510560	Hamburger (with mayonnaise)	655	1.00	290
51150000	Bread roll (white, soft)	145	1.00	52
26137190	Salmon (smoked)	15	0.50	13
71201020	Potato chips	59	0.91	12
71401030	French fries	306	0.95	98
75506010	Mustard	7	0.54	10
91705030	Kit Kat	78	1.00	15
11422000	Yogurt (flavored and lowfat milk)	156	0.99	184
92410510	Soft drink (caffeine free)	49	0.99	122

Day 2

**Food Code**		**Calories (kcal)**	**FPro**	**Grams**
63201010	Blackberries (raw)	11	0.01	26
63219020	Raspberries (raw)	10	0.00	19
92101000	Coffee (regular)	7	0.00	696
57230000	Cereal (Grape-Nuts)	312	1.00	87
53209000	Cookie (chocolate)	206	1.00	44
53112100	Cake	382	1.00	141
58106225	Pizza (cheese)	622	1.00	234
11423000	Yogurt (flavored and nonfat milk)	149	0.84	184

Figure S13: **Dietary recalls for Individual $\mathcal{A}$ (SEQN-ID 68484) and Individual $\mathcal{B}$ (SEQN-ID 59440) annotated in Figure 3a-c.**

3.4 Water Consumption in NHANES

To calculate $iFPro_{WG}$ (Eq. S6), we removed four food codes regarding water consumption, as their reporting through different NHANES cycles showed inconsistencies. Indeed, we noticed that "Water as an ingredient" stopped being tracked since NHANES 2011-2012 (Figure S14 A), and the consumption of tap and bottled water started being recorded since NHANES 2003-2004 (Figures S14B and C). These inconsistencies would have affected our analysis of the pooled cohorts.

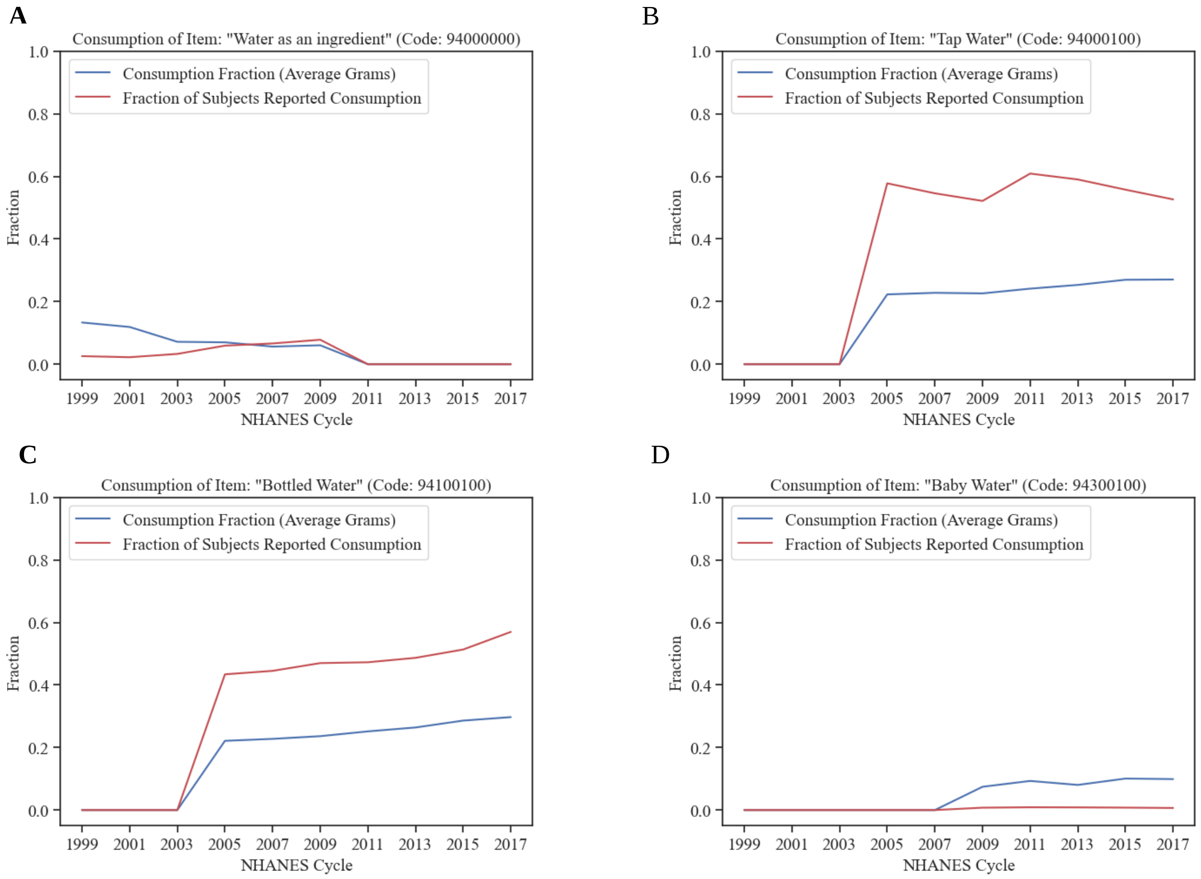

Figure S14: **Water consumption in survey data**. (**a**) "Water as an ingredient" is no longer tracked since NHANES 2011-2012. (**b-c**) Bottled and tap water have been tracked since NHANES 2005-2006, hence this might introduce inconsistency when combining NHANES 1999-2004 cohorts with their succeeding cohorts. (**d**) The consumption of baby water has been tracked since NHANES 2009-2010.

3.5 Relation between $iFPro_{WC}$ and WWEIA Food Categories

By leveraging our calculation of $iFPro_{WC}$ (Eq. 2) over the merged NHANES cohorts between 1999 and 2006 (Section S3.2), we investigated the relation between trends in $iFPro_{WC}$ and fraction of consumed calories in each of the What We Eat in America (WWEIA) food categories [29].

First, for each individual j we calculated the total fraction of calories contributed by food

category g,

$$FC_g^j = \sum_k^{D_j} \frac{c_k^j}{C^j} \delta(WWEIA(k), g), \tag{7}$$

where D_j is the number of dishes consumed by individual j, C^j is the daily total amount of consumed calories, c_k^j is the amount of calories contributed by each food item, and δ indicates the Kronecker delta, whose value is 1 when food k belongs to food category g, and otherwise 0.

We proceeded in calculating the Spearman's rank correlation between $\{iFPro_{WC}^j\}$ and $\{FC_g^j\}$ for each WWEIA class g across the cohort. Once accounted for multiple testing with Bonferroni correction ($\alpha = 0.01$), we found a total of 64 WWEIA categories anti-correlated with $iFPro_{WC}$, and 40 positively correlated (Figure S15).

We further investigated the representation of WWEIA categories in the first quintile Q_1 of $iFPro_{WC}$ ($iFPro_{WC} \leq 0.6908$), compared to the last quintile Q_5 ($iFPro_{WC} \geq 0.8585$). These two sub-populations capture 4,010 individuals each, representing the most divergent dietary patterns in terms of ultra-processed food. The caloric consumption of each WWEIA category across the two sub-groups was tested with the Mann-Whitney U rank test, finding a total of 103 categories significantly changing from Q_1 to Q_5, once corrected for multiple testing (Bonferroni method with $\alpha = 0.01$). To control for "overpowering", as explained in Section S2.6, we calculated the effect size r following [19], and ranked all significant WWEIA categories accordingly (Figure S16A). Overall, the consumption of 21 food groups changes between Q_1 and Q_5 with effect size ≥ 0.1, the baseline for small effect sizes. Among the biggest effect sizes we find "Soft drinks" ($r = 0.4660$), with average caloric fraction 5.61 bigger in Q_5 compared to Q_1, and "Bananas" ($r = 0.2728$), with average caloric fraction 14.07 bigger in Q_1 compared to Q_5 (Figure S16B).

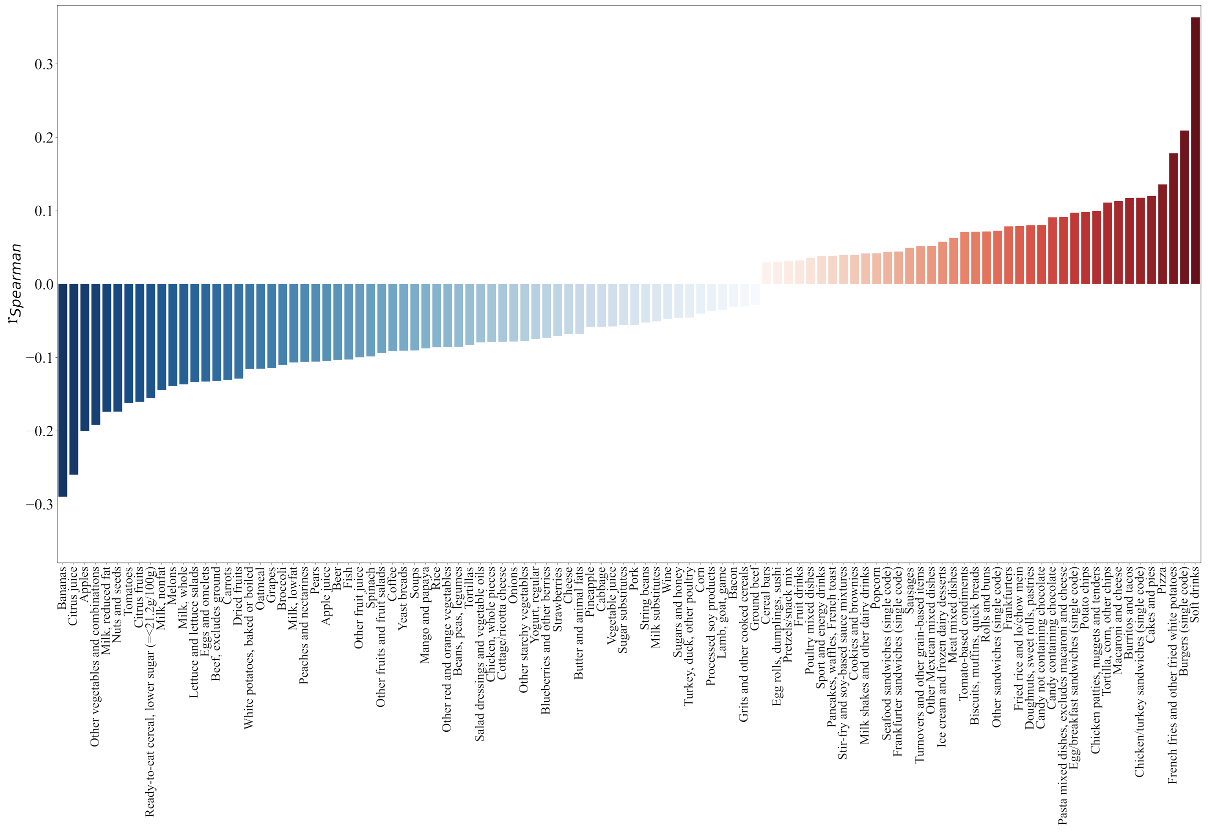

Figure S15: **Spearman's rank correlation between $iFPro_{WC}$ and the caloric fraction contributed by each WWEIA category across NHANES individuals**. 64 WWEIA categories significantly anti-correlate with $iFPro_{WC}$ (blue), while 40 are positively correlated (red). The height of each bar is proportional to the correlation value, and all coefficients are sorted from the strongest anti-correlation to the strongest correlation.

4 Environment-Wide Association Study

Inspired by [30], we performed an Environment-Wide Association Study (EWAS) on the merged NHANES 1999-2006 cohort, to identify environmental factors and disease-related phenotypes associated with $iFPro_{WC}$, $iFPro_{WG}$, and the fraction of calories contributed by manual NOVA 4. To do so, we collected data for 45 exposure modules in [20], and we further added one variable predicting diabetes according to fasting glucose levels ≥126 mg/dL, as advised by the American Diabetes Association [31], two variables predicting metabolic syndrome [32], two assessments of the Framingham Risk Score [33, 34], and the ACC/AHA Risk Score [35], quantifying the 10-year risk of non-fatal myocardial infarction (MI), congestive heart disease (CHD) death, or fatal or nonfatal stroke.

The variables are broadly categorized in two panels (Figure S17A): a health panel, gathering variables describing the overall health of the individuals, from biological age and nutrient biomarkers, to disease phenotypes, and a chemical panel, where we group all chemical exposures measured in blood or urines, linked to pesticides, contaminants, and processing chemical

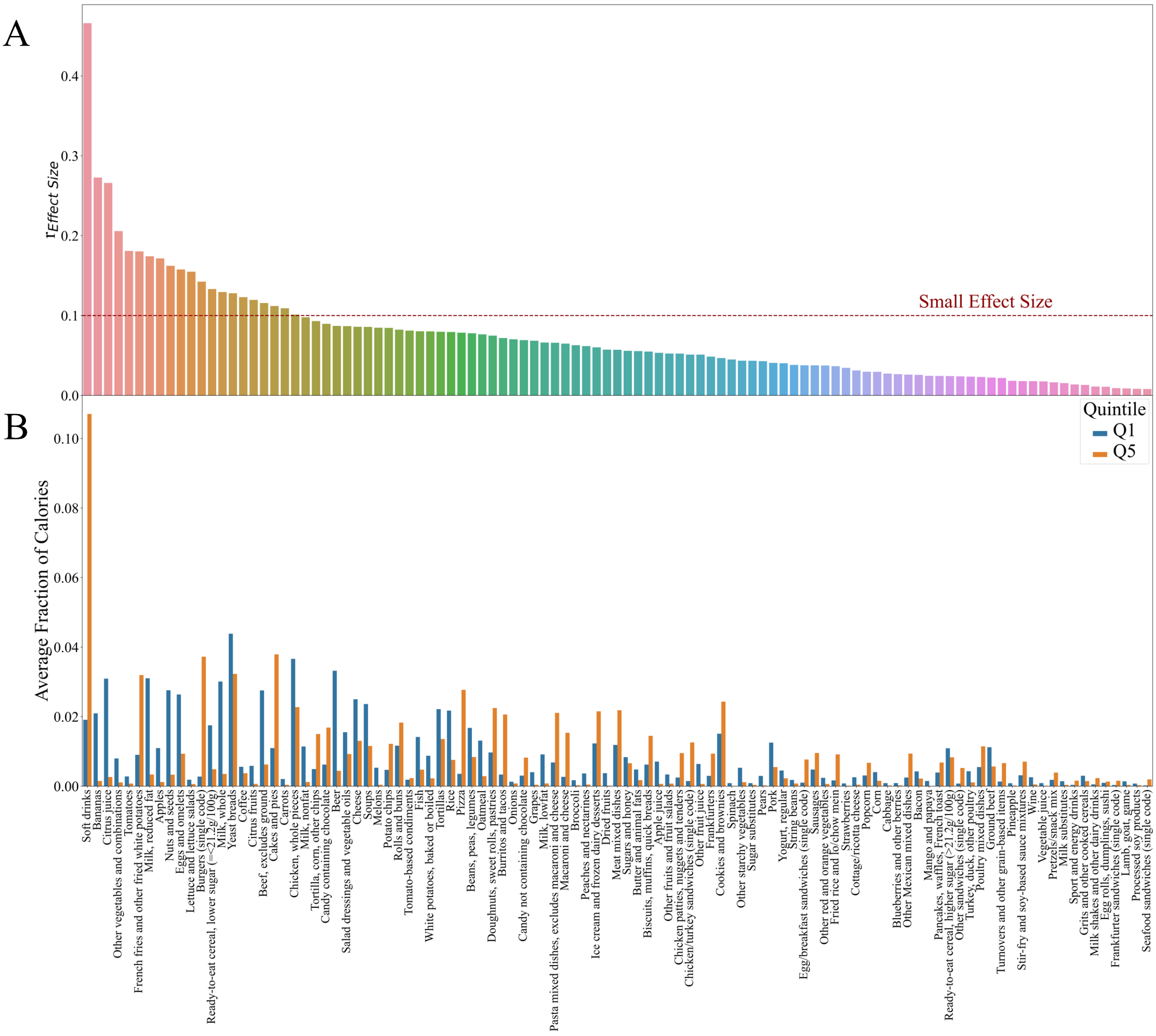

Figure S16: **Comparison of $iFPro_{WC}$ extreme quintiles Q_1 and Q_5.** For individuals in the first quintile Q_1 and in the last quintile Q_5 of $iFPro_{WC}$ we compare the caloric fraction contributed by each WWEIA category. In (a) we estimate the effect size r following [19], where $r \approx 0.1$ is considered a *small* effect, $r \approx 0.3$ a *medium* effect, and $r \approx 0.5$ a *large* effect. The WWEIA food categories surviving multiple testing with Bonferroni correction ($\alpha = 0.01$) are shown in decreasing order of effect size. In (b), following the same order, we show the average fraction of calories in Q_1 and Q_5.

byproducts.

To select the most robust signal, we studied only variables measured in at least two cycles of NHANES. To quantify the statistical associations, we employed survey-weighted generalized linear models, and in particular, linear regression to predict continuous variables, and logistic regression for categorical (Figure S17B). All models were adjusted for age, sex, ethnicity, Body Mass Index (BMI), total-caloric intake, and estimated Socioeconomic Status (SES), as provided by NHANES and consistently with [31] (similar results were obtained with the additional correction for smoking habits). We employed the ‘survey’ statistical package in R to account for the complex survey design of NHANES [26]. We further filtered all categorical and continuous variables lacking a minimum sample size to perform regression analysis. In particular, for continuous variables we considered a ratio between number of covariates and

A

Environmental and Biological Classes		1999–2000	2001–2002	2003–2004	2005–2006	Present in min Two Cohorts
Health Panel	Aging	1	1	0	0	1
Health Panel	Biochemistry	45	45	44	44	47
Health Panel	Blood	20	20	20	20	20
Health Panel	Blood Pressure	4	4	4	4	4
Health Panel	Body Measures	18	18	18	18	18
Health Panel	Cognitive Functioning	2	2	0	0	2
Health Panel	Disease	46	46	46	46	46
Health Panel	Hormone	3	3	3	3	4
Health Panel	Nutrients	10	16	30	18	18
Health Panel	Physical Fitness	15	15	15	1	15
Health Panel	Phytoestrogens	6	6	6	6	6
Exposure Panel	Acrylamide	0	0	2	2	2
Exposure Panel	Dialkyl	7	7	6	0	7
Exposure Panel	Dioxins	6	7	7	0	7
Exposure Panel	Furans	9	10	10	0	10
Exposure Panel	Heavy Metals	15	17	25	22	25
Exposure Panel	Hydrocarbons	14	23	21	0	22
Exposure Panel	PCBs	25	34	38	0	35
Exposure Panel	Perchlorate	0	0	1	0	0
Exposure Panel	Pesticides	29	35	55	23	52
Exposure Panel	Phenols	0	0	3	7	3
Exposure Panel	Phthalates	7	12	13	15	13
Exposure Panel	Polybrominated Ethers	0	0	12	0	0
Exposure Panel	Polyflourochemicals	11	0	12	12	12
Exposure Panel	Volatile Compounds	31	20	38	38	36
	**Total**					405

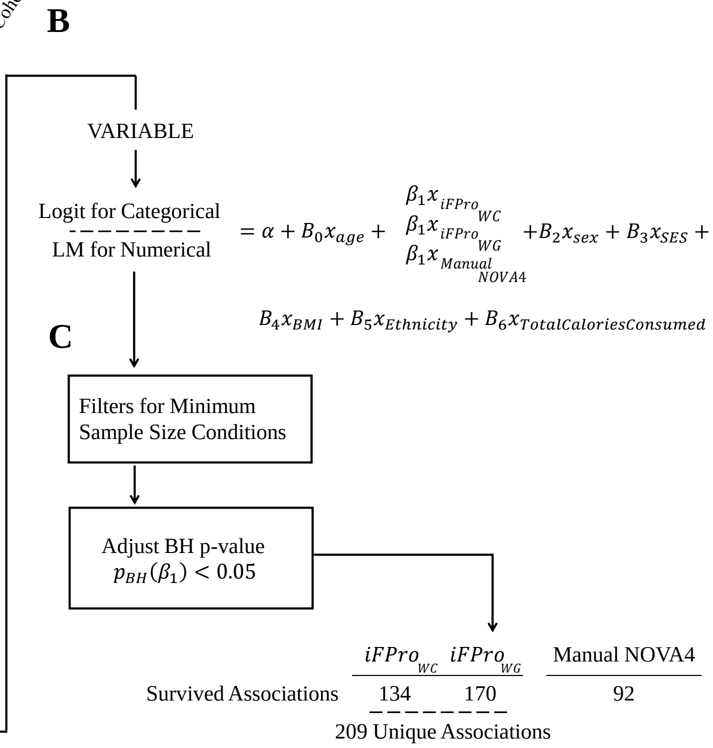

Figure S17: **Overview of the EWAS pipeline on NHANES 1999-2006.** **(a)** For the selected health phenotypes and chemical exposures provided in each cohort of NHANES, we kept only those variables present in at least two cohorts. **(b)** We investigated possible associations between the selected 405 variables in the combined NHANES 1999-2006 cohort using linear regression for continuous variables and logistic regression for categorical variables. **(c)** To account for false discovery rate, we adjusted the p-value of β_1 using Benjamini-Hochberg method with $\alpha = 0.05$.

number of data points $\leq 1/50$, while for categorical we applied a similar threshold for the ratio between number of covariates and number of data points in the smallest category (Figure S17C).

All continuous variables were transformed using Box-Cox transformation or logit function (applied to Framingham and ACC/AHA scores) to stabilize the variance and improve the validity of measures of association [30]. We then standardized all continuous variables, to bring their effect sizes on a similar scale. For multiple linear regression, we used fully standardized regression coefficients, indicating how many standard deviations of change in the dependent variable are associated with one standard deviation increase in the independent variables. For logistic regression, we opted for a partial standardization, acting only on the continuous independent variables, as we wanted to keep a straightforward interpretation of the relation between one standard deviation increase in the Box-Cox transformed $iFPro$ and increase/decrease in disease odds [36].

To account for false discovery rate, we adjusted the p-values corresponding to each score using the Benjamini-Hochberg method with $\alpha = 0.05$. Overall, we find 214 significant tests across the three methodologies, with $iFPro_{WC}$ at 134, $iFPro_{WG}$ at 170, and manual NOVA

4 at 92. The summary of the analysis is presented in Figures S18-S21. A comparison with literature results based on manual NOVA 4 is reported in Table S9.

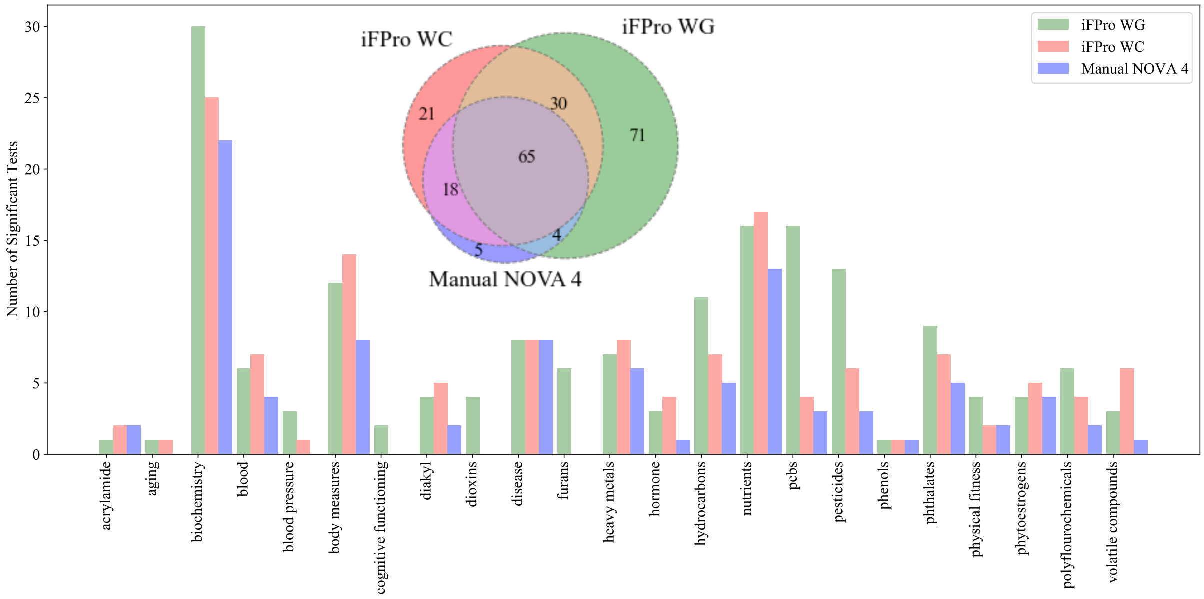

Figure S18: **Number of significant tests surviving multiple testing for $iFPro_{WC}$, $iFPro_{WG}$, and fraction of caloric intake from manual NOVA 4.** For $iFPro_{WG}$ and $iFPro_{WC}$, we find 170 and 134 significant associations, respectively, for a total of 209 unique variables. In comparison, the same analysis using the fraction of calories contributed by manual NOVA 4 finds 92 significant associations, of which 95% is in overlap with $iFPro_{WG}$ and $iFPro_{WC}$. Overall, the total number of variables recovered by the three methodologies is 214. Here we report the results stratified by module.

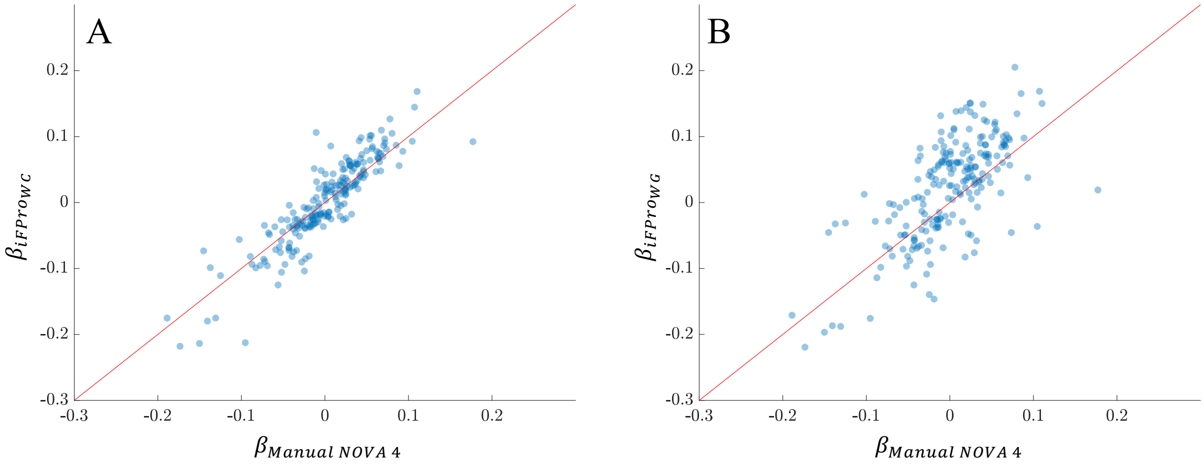

Figure S19: **Comparison of the effect sizes for the significant tests found by $iFPro_{WC}$, $iFPro_{WG}$, and fraction of caloric intake from manual NOVA 4.** Across the 214 significant variables recovered by the three methodologies, we find that **(a)** 70.56% of the times $iFPro_{WC}$ shows bigger effect sizes compared to manual NOVA 4, while **(b)** for $iFPro_{WG}$ the percentage increases to 77.57%.

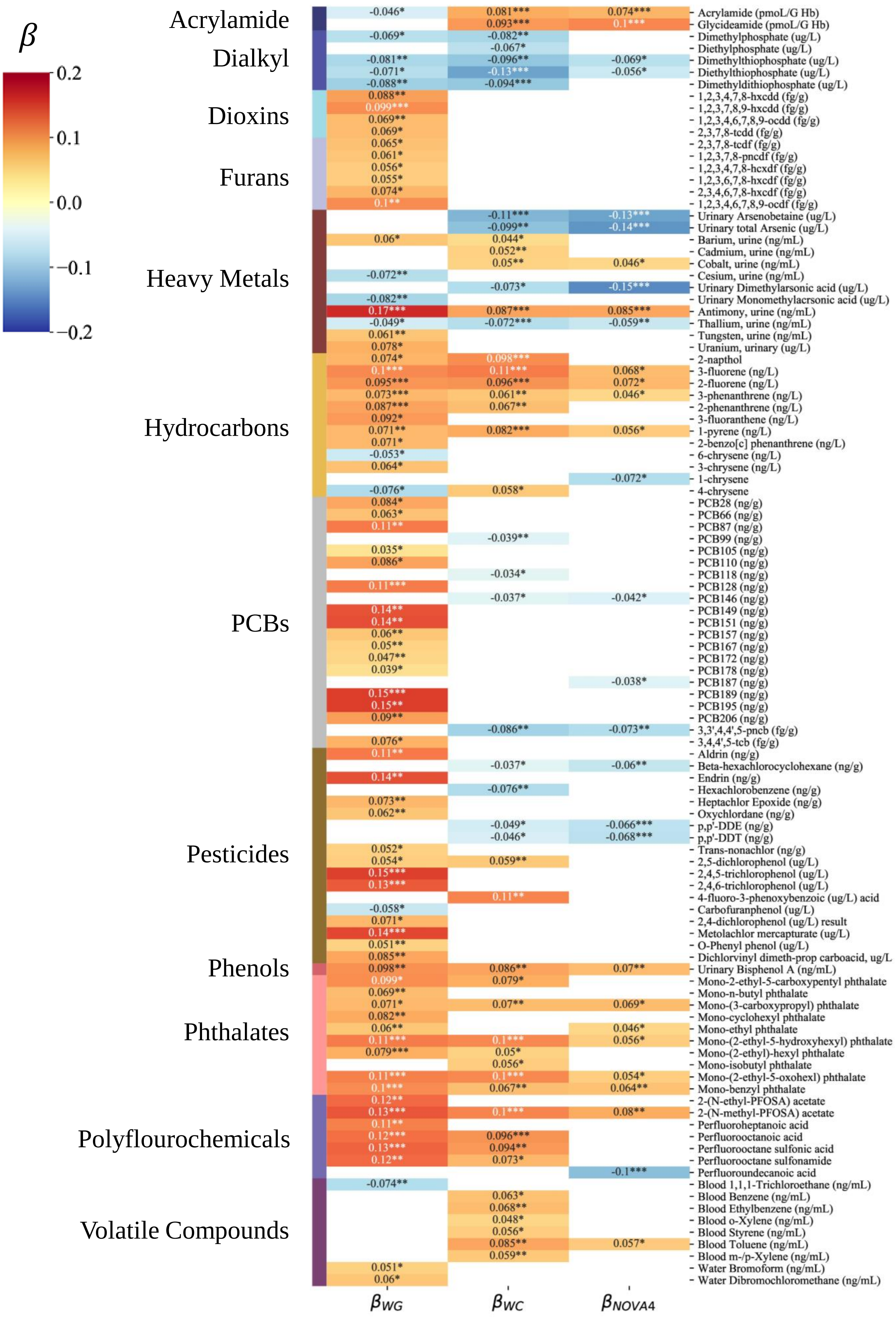
β
0.2
0.1
0.0
−0.1
−0.2
Acrylamide
Dialkyl
Dioxins
Furans
Heavy Metals
Hydrocarbons
PCBs
Pesticides
Phenols
Phthalates
Polyflourochemicals
Volatile Compounds
Acrylamide (pmoL/G Hb)
Glycideamide (pmoL/G Hb)
Dimethylphosphate (ug/L)
Diethylphosphate (ug/L)
Dimethylthiophosphate (ug/L)
Diethylthiophosphate (ug/L)
Dimethyldithiophosphate (ug/L)
1,2,3,4,7,8-hxcdd (fg/g)
1,2,3,7,8,9-hxcdd (fg/g)
1,2,3,4,6,7,8,9-ocdd (fg/g)
2,3,7,8-tcdd (fg/g)
2,3,7,8-tcdf (fg/g)
1,2,3,7,8-pncdf (fg/g)
1,2,3,4,7,8-hcxdf (fg/g)
1,2,3,6,7,8-hxcdf (fg/g)
2,3,4,6,7,8-hxcdf (fg/g)
1,2,3,4,6,7,8,9-ocdf (fg/g)
Urinary Arsenobetaine (ug/L)
Urinary total Arsenic (ug/L)
Barium, urine (ng/mL)
Cadmium, urine (ng/mL)
Cobalt, urine (ng/mL)
Cesium, urine (ng/mL)
Urinary Dimethylarsonic acid (ug/L)
Urinary Monomethylacrsonic acid (ug/L)
Antimony, urine (ng/mL)
Thallium, urine (ng/mL)
Tungsten, urine (ng/mL)
Uranium, urinary (ug/L)
2-napthol
3-fluorene (ng/L)
2-fluorene (ng/L)
3-phenanthrene (ng/L)
2-phenanthrene (ng/L)
3-fluoranthene (ng/L)
1-pyrene (ng/L)
2-benzo[c] phenanthrene (ng/L)
6-chrysene (ng/L)
3-chrysene (ng/L)
1-chrysene
4-chrysene
PCB28 (ng/g)
PCB66 (ng/g)
PCB87 (ng/g)
PCB99 (ng/g)
PCB105 (ng/g)
PCB110 (ng/g)
PCB118 (ng/g)
PCB128 (ng/g)
PCB146 (ng/g)
PCB149 (ng/g)
PCB151 (ng/g)
PCB157 (ng/g)
PCB167 (ng/g)
PCB172 (ng/g)
PCB178 (ng/g)
PCB187 (ng/g)
PCB189 (ng/g)
PCB195 (ng/g)
PCB206 (ng/g)
3,3',4,4',5-pncb (fg/g)
3,4,4',5-tcb (fg/g)
Aldrin (ng/g)
Beta-hexachlorocyclohexane (ng/g)
Endrin (ng/g)
Hexachlorobenzene (ng/g)
Heptachlor Epoxide (ng/g)
Oxychlordane (ng/g)
p,p'-DDE (ng/g)
p,p'-DDT (ng/g)
Trans-nonachlor (ng/g)
2,5-dichlorophenol (ug/L)
2,4,5-trichlorophenol (ug/L)
2,4,6-trichlorophenol (ug/L)
4-fluoro-3-phenoxybenzoic (ug/L) acid
Carbofuranphenol (ug/L)
2,4-dichlorophenol (ug/L) result
Metolachlor mercapturate (ug/L)
O-Phenyl phenol (ug/L)
Dichlorvinyl dimeth-prop carboacid, ug/L
Urinary Bisphenol A (ng/mL)
Mono-2-ethyl-5-carboxypentyl phthalate
Mono-n-butyl phthalate
Mono-(3-carboxypropyl) phthalate
Mono-cyclohexyl phthalate
Mono-ethyl phthalate
Mono-(2-ethyl-5-hydroxyhexyl) phthalate
Mono-(2-ethyl)-hexyl phthalate
Mono-isobutyl phthalate
Mono-(2-ethyl-5-oxohexl) phthalate
Mono-benzyl phthalate
2-(N-ethyl-PFOSA) acetate
2-(N-methyl-PFOSA) acetate
Perfluoroheptanoic acid
Perfluorooctanoic acid
Perfluorooctane sulfonic acid
Perfluorooctane sulfonamide
Perfluoroundecanoic acid
Blood 1,1,1-Trichloroethane (ng/mL)
Blood Benzene (ng/mL)
Blood Ethylbenzene (ng/mL)
Blood o-Xylene (ng/mL)
Blood Styrene (ng/mL)
Blood Toluene (ng/mL)
Blood m-/p-Xylene (ng/mL)
Water Bromoform (ng/mL)
Water Dibromochloromethane (ng/mL)
βWG
βWC
βNOVA4

Figure S20

Figure S20: **Exposure panel**. Each variable reported on the left (e.g., “Acrylamide”) refers to different exposure modules. We report here the standardized β coefficient, quantifying the effect on each exposure when the Box-Cox transformed diet scores increase by one standard deviation over the population. Each variable is color-coded according to β, with positive associations in red, and negative associations in blue. For logistic regressions, p-values are associated with two-sided Wald tests, while for multiple linear regressions, p-values are determined by two-sided t-tests. In white, we annotate the variables that do not survive Benjamini-Hochberg FDR correction with $\alpha = 0.05$ (*** adj p-value < 0.001, ** adj p-value < 0.01, * adj p-value < 0.05)

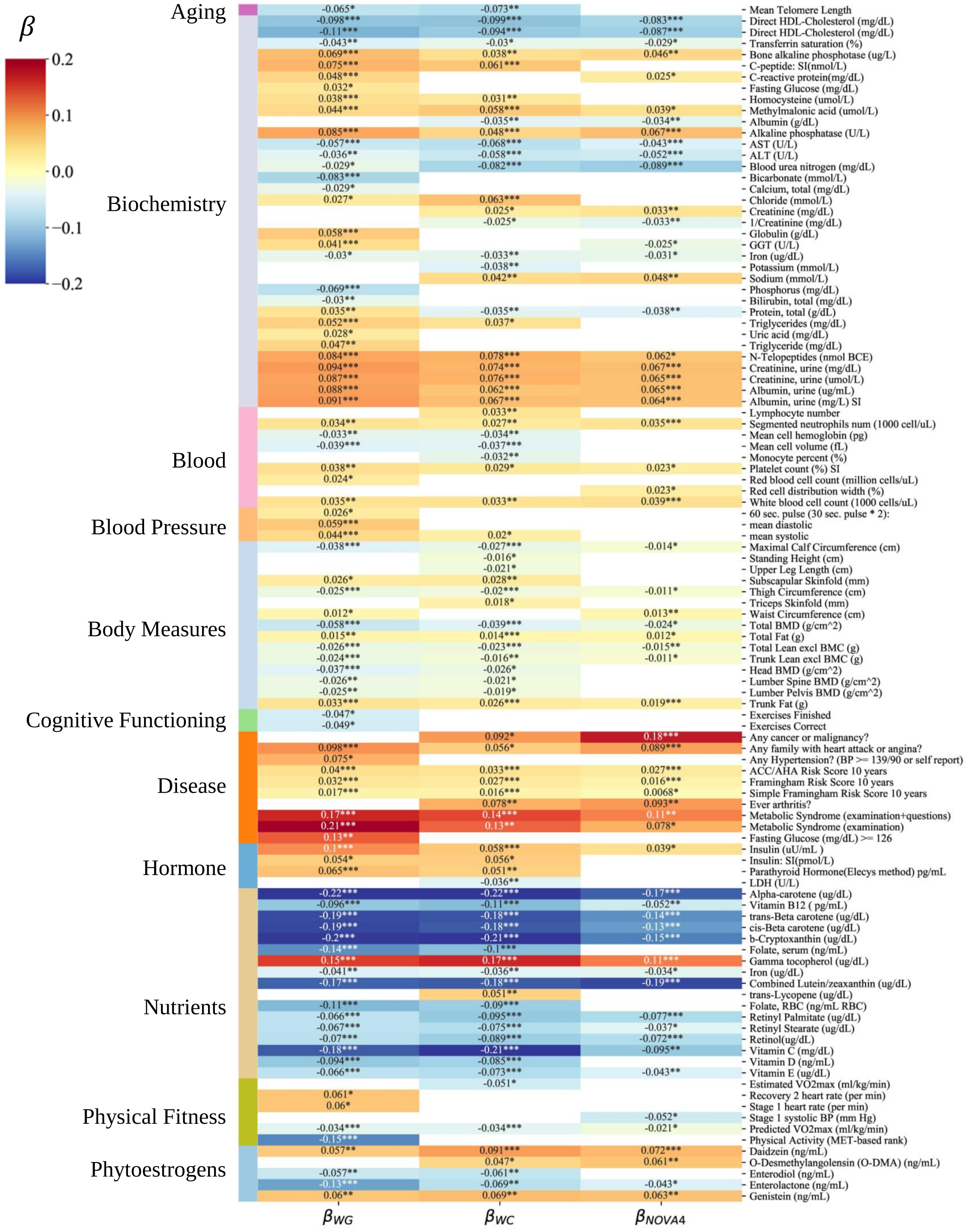

Figure S21

Figure S21: **Health panel**. Each variable reported on the left (e.g., "Aging") refers to different modules of disease phenotypes and measurements assessing the overall health status of each individual. We report here the standardized β coefficient, quantifying the effect on each exposure when the Box-Cox transformed diet scores increase by one standard deviation over the population. Each variable is color-coded according to β, with positive associations in red, and negative associations in blue. Each variable is color-coded according to β, with positive associations in red, and negative associations in blue. For logistic regressions, p-values are associated with two-sided Wald tests, while for multiple linear regressions, p-values are determined by two-sided t-tests. In white, we annotate the variables that do not survive Benjamini-Hochberg FDR correction with $\alpha = 0.05$ (*** adj p-value < 0.001, ** adj p-value < 0.01, * adj p-value < 0.05)

Table S9: A Summary of the Epidemiological Literature on the Discovered Associations with Manual NOVA 4.

**Health or Exposure Panel**	**Paper**	**In Agreement with *iFPro***
Aging	Ultra-processed food consumption and the risk of short telomeres in an elderly population of the Seguimiento Universidad de Navarra (SUN) Project [37] Spain (SUN)	Yes
Disease: Cardiovascular	• Association between ultraprocessed food intake and cardiovascular health in US adults: a cross-sectional analysis of the NHANES 2011–2016 [38] USA (NHANES)<br>• Ultra-processed food intake and risk of cardiovascular disease: prospective cohort study (NutriNet-Santé) [39] France (NutriNet-Santé)<br>• Ultra-processed food consumption is associated with increased risk of all-cause and cardiovascular mortality in the Moli-sani Study [40] Molise, Italy (Moli-sani)	Yes
Disease: Hypertension	Ultra-processed food consumption and the incidence of hypertension in a mediterranean cohort: The seguimiento universidad de navarra project [41] Spain (SUN)	Yes
Continued on next page		

Table S9 – continued from previous page

**Health or Exposure Panel**	**Paper**	**In Agreement with *iFPro***
Disease: Metabolic Syndrome	• Dietary share of ultra-processed foods and metabolic syndrome in the US adult population [42] USA (NHANES)<br>• Diet quality indices in relation to metabolic syndrome in an Indigenous Cree (Eeyouch) population in northern Québec, Canada [43] Canada (Aschii Environment & Health Study)<br>• A minimally processed dietary pattern is associated with lower odds of metabolic syndrome among Lebanese adults [44] Lebanon	Yes
Disease: Cancer	Consumption of ultra-processed foods and cancer risk: results from NutriNet-Santé prospective cohort [45] France (NutriNet-Santé)	Yes
Body Measures: Total Fat	Contribution of ultra-processed foods in visceral fat deposition and other adiposity indicators: Prospective analysis nested in the PREDIMED-Plus trial [46] Spain (PREDIMED-Plus)	Yes

Continued on next page

Table S9 – continued from previous page

**Health or Exposure Panel**	**Paper**	**In Agreement with** ***iFPro***
Body Measures: Waist Circumference	• Ultra-processed food consumption and excess weight among US adults [47] USA (NHANES)<br>• Consumption of ultra-processed food and obesity: cross sectional results from the Brazilian Longitudinal Study of Adult Health (ELSA-Brasil) cohort (2008–2010) [48] Brazil (ELSA)<br>• Ultra-processed food consumption and indicators of obesity in the United Kingdom population (2008-2016) [49] UK (NDNS)	Yes
Nutrients	• The share of ultra-processed foods and the overall nutritional quality of diets in the US: evidence from a nationally representative cross-sectional study [50] USA (NHANES)<br>• Impact of ultra-processed foods on micronutrient content in the Brazilian diet [51] Brazil (HBS)	Yes
Biochemistry: C-Reactive Protein	Association between consumption of ultra-processed foods and serum C-reactive protein levels: cross-sectional results from the ELSA-Brasil study [52] Brazil (ELSA)	Yes
Phytoestrogens	Association between dietary share of ultra-processed foods and urinary concentrations of phytoestrogens in the US [53] USA (NHANES)	Yes
Vegetarian Diet: Adverse Effects of ultra-processing	Consumption of Ultra-Processed Foods by Pesco-Vegetarians, Vegetarians, and Vegans: Associations with Duration and Age at Diet Initiation [54] France (NutriNet-Santé)	Yes

Continued on next page

Table S9 – continued from previous page

**Health or Exposure Panel**	**Paper**	**In Agreement with $iFPro$**
Phthalates and Phenols	• Ultra-processed food consumption and exposure to phthalates and bisphenols in the US National Health and Nutrition Examination Survey, 2013–2014 [55] USA (NHANES)<br>• Association between dietary contribution of ultra-processed foods and urinary concentrations of phthalates and bisphenol in a nationally representative sample of the US population aged 6 years and older [56] USA (NHANES)	Yes
Acrylamides	Association between Heat-Induced Chemical Markers and Ultra-Processed Foods: A Case Study on Breakfast Cereals [57] Spanish supermarkets	Yes

5 Food Substitution

The observed variability of $FPro$ for categories of foods similarly consumed in the population (Figure 2E), combined with the EWAS results, quantifying the effect of processed diet on disease risk, suggests a systematic way to implement food substitution and predict its relevance in terms of health indicators [58, 59]. With this goal, we classified all foods consumed by the pooled cohort of 20,047 individuals in NHANES 1999-2006, according to WWEIA [60]. For substitution purposes, the relevance of food k in individual j's diet can be quantified as

$$r_k^j = f_C^{(k,j)}(FPro_k - FPro_{min\ WWEIA(k)}), \tag{8}$$

where $f_C^{(k,j)}$ is the fraction of calories contributed by food k to the dietary profile, while $FPro_{min\ WWEIA(k)}$ refers to the food with the lowest $FPro$ within the same WWEIA category of food k. By picking the suggested foods in the original WWEIA classes reported by each individual, we aim to minimally perturb her habits, to maximize the compliance to the new dietary regime. Moreover, as Eq. S8 offers a heuristic to identify which food to prioritize, the overall level of processing is reduced in a minimal number of steps.

The impact of substituting M foods on disease risk is measured in terms of odds ratio (OR), that quantifies the odds of disease occurring when adopting the optimized diet, compared to the original choices. We estimate OR as

$$OR^j_{(Sub\ vs\ Orig)} = e^{\beta_1[(iFPro^j_{WC} - \sum_{m=1}^{M} r^j_m)^t - (iFPro^j_{WC})^t]}, \tag{9}$$

where β_1 is the effect size describing the strength of the association between $iFPro$ and disease onset, all $\{r^j_m\}$ follow from Eq. S8, and with the superscript t we denote the function-composition of Box-Cox transformation followed by z-score with parameters estimated in the original population.

For continuous variables y like vitamin B_{12}, vitamin C, and bisphenol A, the impact of substituting M foods on individual j's diet is quantified by

$$\frac{y^j_{Sub}}{y^j_{Orig}} = \frac{\{(y^j_{Orig})^t + \beta_1[(iFPro^j_{WC} - \sum_{m=1}^{M} r^j_m)^t - (iFPro^j_{WC})^t]\}^{-t}}{y^j_{Orig}}, \tag{10}$$

where with the superscript $-t$ we refer to the inverse function-composition of Box-Cox transformation followed by standardization (first invert z-score, then Box-Cox).

6 Open Food Facts

Open Food Facts is a free, crowd-sourced world-wide database of food products, with nutrition facts and ingredient list [61]. We collected 233,831 nutritional records from their website, corresponding to 168,681 product ids, annotated with NOVA labels according to a heuristic described at [62]. This database also compiled an extensive list of food additives that gave us the possibility to quantify the number of additives per product. Similarly to the cross-validation explained in Section S2.1, we trained and validated two models on the same 5-fold partition: (1) standard FoodProX leveraging the logarithm of 11 nutrients used as baseline, (2) FoodProX with an additional input feature capturing the number of additives in each food. The baseline feature panel includes protein, fat, total carbohydrate, sugars, dietary fiber, calcium, iron, sodium, cholesterol, saturated fat, and trans fat. We removed vitamins A and C from the analysis, given the high number of not available values (NAs). Hence, the cross-validation was run on a selection of 228,689 records, with no NAs in any feature.

Overall, we find commendable performances in both scenarios, with higher AUC and AUP when additives are included in the model (see Table S10). In particular, model (2) outperforms

model (1) in assessing NOVA 3. We derived $FPro_1$ and $FPro_2$ following Eq. 1, finding they highly correlate with each other ($\rho_{Spearman} = 0.9017$), and they both correlate well with the number of additives ($\rho_{Spearman}(FPro_1, n_{additives}) = 0.6726$, $\rho_{Spearman}(FPro_2, n_{additives}) = 0.8240$). This result is encouraging, as details regarding ingredient lists and additives are seldom if ever available in current food composition databases.

To better understand the role of additives in discriminating NOVA classes, we trained and validated a random forest taking the number of additives as the only input, over the same train-test split used for model (1) and (2). We find AUC equal to 0.859923 ± 0.000485 for NOVA 1, 0.832023 ± 0.003375 for NOVA 2, 0.820256 ± 0.000693 for NOVA 3, and 0.907307 ± 0.000609 for NOVA 4. Compared to model (2), the performances in terms of AUC drop of 13.36% for NOVA 1, 15.77% for NOVA 2, 15.03% for NOVA 3, and 7.25% for NOVA 4.

The lack of information on nutrient amounts strongly affects the precision-recall curve, increasing disproportionately the number of false positives predicted for NOVA 1, 2, and 3. Indeed, we find AUP equal to 0.288799 ± 0.000930 for NOVA 1, 0.026227 ± 0.000498 for NOVA 2, 0.439202 ± 0.001269 for NOVA 3, and 0.941548 ± 0.000350 for NOVA 4. The values for class NOVA 1, 2, and 3 are close to the proportion of examples labeled as 1, 2, and 3 in the training, suggesting that the model's behavior is close to a random classifier. Compared to model (2), the performances in terms of AUP drop of 70.01% for NOVA 1, 97.05% for NOVA 2, 50.96% for NOVA 3, and 4.77% for NOVA 4.

Overall, these results suggest that the number of additives is a good predictor of ultra-processed food as defined by NOVA, but it misses the processing nuances represented in the other NOVA classes.

Table S10: **AUC and AUP for the four NOVA classes in Open Food Facts**. For model (1) and (2) we report the average and standard deviation of AUC (A) and AUP (B) over the stratified 5-folds.

(A)

	NOVA 1	NOVA 2	NOVA 3	NOVA 4
Average AUC 11 Nutrients	0.987989	0.986044	0.932033	0.950768
Std AUC 11 Nutrients	0.000647	0.004472	0.001525	0.00086
Average AUC 11 Nutrients + Additives	0.992553	0.987785	0.965301	0.978242
Std AUC 11 Nutrients + Additives	0.000327	0.004682	0.000962	0.000683

(B)

	NOVA 1	NOVA 2	NOVA 3	NOVA 4
Average AUP 11 Nutrients	0.949175	0.85327	0.828676	0.972467
Std AUP 11 Nutrients	0.002694	0.016713	0.002751	0.000712
Average AUP 11 Nutrients + Additives	0.962916	0.890253	0.895639	0.988722
Std AUP 11 Nutrients + Additives	0.001583	0.013054	0.002983	0.000388

7 Data Quality and Future Directions

- **Data Sample and Variability**. The quality of the training data influences our analysis and the interpretation of *FPro*. Indeed, all machine learning and AI models become feasible only when extensive labeled datasets are available, and on top of that, they provide reliable results when the training data samples the real world in an exhaustive way, avoiding over-fitting.

  A single cycle of FNDDS, as well as SR-Legacy, reports representative nutritional average values for each food/drink, which do not capture the variability due to factors such as recipe variations, production methods, soil quality, and storage time. The training data would then benefit from multiple instances of the same food, helping capture the natural variability in nutrient content, and reduce over-fitting [63]. This is why we have investigated how nutritional values for the same food code change through different FNDDS cycles, and how integrating additional variability is affecting FoodProX and *FPro* in assessing "unseen foods" (see *Assesment of FPro robustness* below). We envision that Foundation Foods, the new USDA food composition dataset available at FoodData Central, will be a great asset to improve the current data training of *FPro*, once it will increase its coverage to more than 140 foods. Indeed, Foundation Foods includes individual sample measurements behind the nutrient mean values that populate the other databases, and metadata reporting the number of samples, location, time-stamps, analytical methods used, and, additionally, if available, cultivar and production practices.

  A further way to improve how machine learning algorithms and AI characterize food data is to study the statistical properties of nutrient distributions in the food supply and make sure that the training data sample them exhaustively. In Menichetti et al. [9] we observe how the variability of nutrient concentrations in food, despite covering several orders of magnitudes, follows a well-defined functional form, related to the lognormal distribution. This is the reason why we log-transformed all nutrient measures before performing the classification, as this is the natural scale of the nutrient fluctuations. Accounting for the variability driven by chemical reactions in the ingredients, as well as for the variability derived from the complete food production chain, plays a major role in successfully capturing associations between food intake and disease phenotypes [64].

  Beyond nutrient variability, the training data would benefit from a better representation of different food groups, as in the case of raw meat products, important ingredients in many recipes. This poor representation in the dataset is somehow expected, as FNDDS is designed by the USDA to provide food composition data for foods and beverages reported

in the dietary component of NHANES, and raw meat is not commonly consumed by the American population. Better coverage of a heterogeneous collection of food groups would also improve the accuracy of the algorithm when facing different national food composition databases, beyond the data collected by the USDA.

Finally, we stress that all potential errors affecting food composition data should generally be random with respect to the machine learning algorithm and the manual NOVA classification that developed $FPro$, which should only cause attenuation of classification accuracy rather than bias, and attenuation toward the null of all epidemiological outcomes. Thus, our findings would further improve with a more accurate database.

- **Requirements for Branded Products**. While in this study we focus on survey data, our model based on nutritional values can easily work on different food and cohort databases, as proven by our analysis of over 50,000 products collected from major grocery store websites [65]. When facing real-world food data, we have to account for the regulations introduced by government agencies, like the FDA. For instance, in the nutrition facts label nutrients are classified into 3 different classes, characterized by different standards regarding the agreement between the declared value on the label and the actual values in the sampled food [66]. For nutrients like sugars, total fat, saturated fat, cholesterol, and sodium, the label is considered compliant if the nutrient content of the sampled product is up to 20% above the value declared on the label. Consequently, when relying on the nutrition facts of branded products, $FPro$ should be reported with confidence intervals determined by randomly altering the nutrient content according to FDA regulations.
- **Size and Composition of the Nutrient Panel**. The computation of $FPro$ adapts to different sets of nutrients, allowing us to accurately classify food from limited nutrient information. However, the chemical information currently available to train our algorithm is limited by the resolution of food databases. Indeed, many chemicals like acrylamide, ammonium sulfate, azodicarbonamide, butylated hydroxyanisole, and furans, associated with the preparation and preservation of food, are not tracked by national agencies. The lack of quantification of these chemicals becomes even more striking once we acknowledge their impact on human health [67–71]. With a higher number of chemicals available for all foods, we could aim for an unsupervised classification of food processing, eliminating the need for supervised manual curation. Currently, our analysis in Section S1.5 shows that an unsupervised hierarchical clustering of foods, leveraging the widest nutrient panel

available in FNDDS, is not able to independently reproduce the four NOVA classes. It is possible, however, that the addition of chemical measurements that pertain to processing signatures could further improve the current results, leading to a purely chemically driven classification of food processing.

- **Assessment of $FPro$ robustness**. We aim to test the robustness of $FPro$ by measuring the extent of its variations when re-formulations of the same product are created and measured experimentally. This analysis is currently hindered by limited data availability. However, we were able to test how nutrient variability impacts $FPro$ when comparing the same food code in different cycles of FNDDS. Indeed, while FNDDS does not capture sample variability within the same cycle (e.g., 2009-2010), it documents the nutrient variability affecting the same food through the years, as a function of the specific sample or technique chosen to estimate the representative nutritional average. In FoodProXonFNDDS.xlsx we report the nutrient profile of 5,632 foods, present in both FNDDS 2009-2010 and 2015-2016, and how their $FPro$ changed according to the variations in their nutrient content. For all foods we estimate the number of nutrients that changed between the two editions of FNDDS, accounting for the different rounding approximations affecting each nutrient. In presence of one or more nutrients with maximal variation between 10% and 50%, we observe a median $\Delta FPro$=0.001556 (Q_1=0.000222, Q_3= 0.004764). Allowing significantly bigger fluctuations, as in case of one or more nutrients with maximal variation between 10% and 1000%, we observe a median $\Delta FPro$= 0.003312 (Q_1= 0.000722, Q_3= 0.011310).

- **Limitations of Population Surveys**. To test the prediction power of $FPro$ and $iFPro$ for several health phenotypes and chemical exposures, we leveraged the rich panel of analyses collected in NHANES, a population survey that captures dietary intake with 24-hour recalls. A limitation of the 24-hour recall is its reliance on memory, both for identification of foods eaten as well as for quantification of portion sizes, and the need for highly trained interviewers. Although reliance on the participant's memory leaves room for measurement error, skilled interviewers can produce highly detailed and useful nutritional data comparable to a dietary record [24]. 24-hour recalls are also affected by random within-person error, typified by the day-to-day fluctuation in dietary intake. Random within-person error tends to decrease correlation and regression coefficients towards zero and to bias relative risks toward one. Multiple days of intake per individual permit an estimate of within-person day-to-day variability, and it is usually statistically more efficient to increase the number of individuals in the sample rather than increase the number of days beyond two per individual [72]. For this reason, NHANES added a second day of

dietary intake starting with NHANES 2003 [24]. Despite these limitations, 24-hour recalls are widely used in epidemiology as their statistical issues are well understood, and they were used to rigorously evaluate HEI-2015, a gold-standard non-data-driven dietary score for the epidemiological community [73].

Supplementary References

[1] FDA Nutrition Facts. https://www.fda.gov/media/99331/download.

[2] USDA FoodData Central. https://fdc.nal.usda.gov/.

[3] Ahuja, J. *et al.* USDA Food and Nutrient Database for Dietary Studies, 5.0. U.S. Department of Agriculture, Agricultural Research Service, Food Surveys Research Group, Beltsville, MD. http://www.ars.usda.gov/ba/bhnrc/fsrg (2012).

[4] Sebastian, R. S. *et al.* Flavonoid Values for USDA Survey Foods and Beverages 2007–2010. U.S. Department of Agriculture, Agricultural Research Service, Food Surveys Research Group, Beltsville, MD. http://www.ars.usda.gov/nea/bhnrc/fsrg (2016).

[5] USDA National Nutrient Database for Standard Reference (SR). URL https://data.nal.usda.gov/dataset/usda-national-nutrient-database-standard-reference-legacy-release.

[6] USDA FoodData Central (FDC) . URL https://fdc.nal.usda.gov/.

[7] Steele, E. M. *et al.* Ultra-processed foods and added sugars in the US diet: Evidence from a nationally representative cross-sectional study. *BMJ Open* **6**, 1–8 (2016).

[8] Database of Flavonoid Values for USDA Food Codes 2007-2010 and Flavonoid Intake Data Files from What We Eat in America (WWEIA), National Health and Nutrition Examination Survey (NHANES) 2007-2010 . URL https://www.ars.usda.gov/northeast-area/beltsville-md-bhnrc/beltsville-human-nutrition-research-center/food-surveys-research-group/docs/fndds-flavonoid-database/.

[9] Menichetti, G. & Barabási, A.-L. Nutrient concentrations in food display universal behaviour. *Nature Food 2022 3:5* **3**, 375–382 (2022). URL https://www.nature.com/articles/s43016-022-00511-0.

[10] Langfelder, P., Zhang, B. & Horvath, S. Defining clusters from a hierarchical cluster tree: The Dynamic Tree Cut package for R. *Bioinformatics* **24**, 719–720 (2008).

[11] Vinh, N. X., Epps, J. & Bailey, J. Information theoretic measures for clusterings comparison: Variants, properties, normalization and correction for chance. *Journal of Machine Learning Research* **11**, 2837–2854 (2010).

[12] Chawla, N. V., Bowyer, K. W., Hall, L. O. & Kegelmeyer, W. P. SMOTE: Synthetic minority over-sampling technique. *Journal of Artificial Intelligence Research* **16**, 321–357 (2002). 1106.1813.

[13] Parr, T., Turgutlu, K., Csiszar, C. & Howard, J. Permutation Feature Importance. URL github.com/parrt/random-forest-importances.

[14] Strobl, C., Boulesteix, A. L., Zeileis, A. & Hothorn, T. Bias in random forest variable importance measures: Illustrations, sources and a solution. *BMC Bioinformatics* **8**, 1–21 (2007). URL https://link.springer.com/articles/10.1186/1471-2105-8-25https://link.springer.com/article/10.1186/1471-2105-8-25.

[15] Lundberg, S. M. & Lee, S.-I. A Unified Approach to Interpreting Model Predictions. *Advances in Neural Information Processing Systems 30* 4765–4774 (2017). URL http://papers.nips.cc/paper/7062-a-unified-approach-to-interpreting-model-predictions.pdf. 1705.07874.

[16] Kapur, J. N. *Maximum-Entropy Models in Science and Engineering.* (Wiley, 1989).

[17] Monteiro, C. A. *et al.* NOVA. The star shines bright. *World Nutrition* **7**, 28–38 (2016).

[18] Post Shredded Wheat: WHEAT 'N BRAN. URL https://www.postshreddedwheat.com/products/wheat-n-bran/.

[19] Mann-Whitney Test for Independent Samples. URL http://www.real-statistics.com/non-parametric-tests/mann-whitney-test/.

[20] Patel, C. J. *et al.* A database of human exposomes and phenomes from the us national health and nutrition examination survey. *Scientific Data* **3**, 160096 (2016). URL https://doi.org/10.1038/sdata.2016.96.

[21] Measuring guides for the dietary recall interview. https://www.cdc.gov/nchs/nhanes/measuring_guides_dri/measuringguides.htm. Accessed: 2021-09-20.

[22] Reedy, J. *et al.* Evaluation of the healthy eating index-2015. *Journal of the Academy of Nutrition and Dietetics* **118**, 1622–1633 (2018). URL https://www.sciencedirect.com/science/article/pii/S2212267218308360.

[23] Nhanes phone follow-up dietary interviewer procedures manual. https://www.cdc.gov/nchs/data/nhanes/nhanes_09_10/phone_follow_up_dietary_procedures_manual_mar_2010.pdf (2010). Accessed: 2021-09-20.

[24] Satija, A., Yu, E., Willett, W. C. & Hu, F. B. Understanding Nutritional Epidemiology and Its Role in Policy. *Advances in Nutrition* **6**, 5–18 (2015). URL `https://doi.org/10.3945/an.114.007492`. `https://academic.oup.com/advances/article-pdf/6/1/5/23880586/5.pdf`.

[25] NHANES Survey Methods and Analytic Guidelines. URL `https://wwwn.cdc.gov/nchs/nhanes/analyticguidelines.aspx`.

[26] Lumley, T. survey: analysis of complex survey samples (2020). R package version 4.0.

[27] Healthy Eating Index (HEI) . URL `https://www.fns.usda.gov/resource/healthy-eating-index-hei`.

[28] National cancer institute. developing the healthy eating index. bethesda, md: National cancer institute. https://epi.grants.cancer.gov/hei/developing.html. 2020 (accessed September 1, 2020).

[29] Rhodes, D. G., Adler, M. E., Clemens, J. C. & Moshfegh, A. J. What we eat in America food categories and changes between survey cycles. *Journal of Food Composition and Analysis* **64**, 107–111 (2017). URL `http://dx.doi.org/10.1016/j.jfca.2017.07.018`.

[30] Milanlouei, S. *et al.* A systematic comprehensive longitudinal evaluation of dietary factors associated with acute myocardial infarction and fatal coronary heart disease. *Nature Communications* **11**, 1–14 (2020). URL `https://doi.org/10.1038/s41467-020-19888-2`.

[31] Patel, C. J., Bhattacharya, J. & Butte, A. J. An environment-wide association study (EWAS) on type 2 diabetes mellitus. *PLoS ONE* **5** (2010).

[32] Moore, J., Chaudhary, N. & Akinyemiju, T. Metabolic syndrome prevalence by race/ethnicity and sex in the united states, national health and nutrition examination survey, 1988–2012. *Prev Chronic Dis* (2017).

[33] D'Agostino, R. B. *et al.* General cardiovascular risk profile for use in primary care: The Framingham heart study. *Circulation* **117**, 743–753 (2008).

[34] Castro, V. *CVrisk: Compute Risk Scores for Cardiovascular Diseases* (2021). URL `https://github.com/vcastro/CVrisk`. R package version 1.1.0.9000.

[35] Goff, D. C. *et al.* 2013 ACC/AHA guideline on the assessment of cardiovascular risk: A report of the American college of cardiology/American heart association task force on practice guidelines. *Circulation* **129**, 49–73 (2014).

[36] Menard, S. Standards for standardized logistic regression coefficients. *Social Forces* **89**, 1409–1428 (2011).

[37] Alonso-Pedrero, L. *et al.* Ultra-processed food consumption and the risk of short telomeres in an elderly population of the Seguimiento Universidad de Navarra (SUN) Project. *The American journal of clinical nutrition* **111**, 1259–1266 (2020). URL https://academic.oup.com/ajcn/article/111/6/1259/5824715.

[38] Zhang, Z., Jackson, S. L., Martinez, E., Gillespie, C. & Yang, Q. Association between ultraprocessed food intake and cardiovascular health in us adults: a cross-sectional analysis of the nhanes 2011–2016. *The American Journal of Clinical Nutrition* **113**, 428–436 (2020). URL https://doi.org/10.1093/ajcn/nqaa276. https://academic.oup.com/ajcn/article-pdf/113/2/428/36170430/nqaa276.pdf.

[39] Srour, B. *et al.* Ultra-processed food intake and risk of cardiovascular disease: prospective cohort study (nutrinet-santé). *BMJ* **365** (2019). URL https://www.bmj.com/content/365/bmj.l1451. https://www.bmj.com/content/365/bmj.l1451.full.pdf.

[40] Bonaccio, M. *et al.* Ultra-processed food consumption is associated with increased risk of all-cause and cardiovascular mortality in the Moli-sani Study. *The American Journal of Clinical Nutrition* **113**, 446–455 (2020). URL https://doi.org/10.1093/ajcn/nqaa299. https://academic.oup.com/ajcn/article-pdf/113/2/446/36170506/nqaa299.pdf.

[41] De Deus Mendonça, R. *et al.* Ultra-processed food consumption and the incidence of hypertension in a mediterranean cohort: The seguimiento universidad de navarra project. *American Journal of Hypertension* **30**, 358–366 (2017). URL https://pubmed.ncbi.nlm.nih.gov/27927627/.

[42] Martínez Steele, E., Juul, F., Neri, D., Rauber, F. & Monteiro, C. A. Dietary share of ultra-processed foods and metabolic syndrome in the us adult population. *Preventive Medicine* **125**, 40–48 (2019). URL https://www.sciencedirect.com/science/article/pii/S0091743519301720.

[43] Lavigne-Robichaud, M. *et al.* Diet quality indices in relation to metabolic syndrome in an indigenous cree (eeyouch) population in northern québec, canada. *Public Health Nutrition* **21**, 172–180 (2018).

[44] Nasreddine, L. *et al.* A minimally processed dietary pattern is associated with lower odds of metabolic syndrome among lebanese adults. *Public Health Nutrition* **21**, 160–171 (2018).

[45] Fiolet, T. *et al.* Consumption of ultra-processed foods and cancer risk: results from nutrinet-santé prospective cohort. *BMJ* **360** (2018). URL https://www.bmj.com/content/360/bmj.k322. https://www.bmj.com/content/360/bmj.k322.full.pdf.

[46] Konieczna, J. *et al.* Contribution of ultra-processed foods in visceral fat deposition and other adiposity indicators: Prospective analysis nested in the predimed-plus trial. *Clinical Nutrition* (2021). URL https://www.sciencedirect.com/science/article/pii/S0261561421000297.

[47] Juul, F., Martinez-Steele, E., Parekh, N., Monteiro, C. A. & Chang, V. W. Ultra-processed food consumption and excess weight among us adults. *British Journal of Nutrition* **120**, 90–100 (2018).

[48] Silva, F. M. *et al.* Consumption of ultra-processed food and obesity: cross sectional results from the brazilian longitudinal study of adult health (elsa-brasil) cohort (2008–2010). *Public Health Nutrition* **21**, 2271–2279 (2018).

[49] Rauber, F. *et al.* Ultra-processed food consumption and indicators of obesity in the united kingdom population (2008-2016). *PLOS ONE* **15**, 1–15 (2020). URL https://doi.org/10.1371/journal.pone.0232676.

[50] Martínez Steele, E., Popkin, B. M., Swinburn, B. & Monteiro, C. A. The share of ultra-processed foods and the overall nutritional quality of diets in the us: evidence from a nationally representative cross-sectional study. *Population Health Metrics* **15**, 6 (2017). URL https://doi.org/10.1186/s12963-017-0119-3.

[51] Louzada, M. L. d. C. *et al.* Impact of ultra-processed foods on micronutrient content in the Brazilian diet. *Revista de saude publica* **49** (2015). URL https://doi.org/10.1590/S0034-8910.2015049006211.

[52] Lopes, A. E. d. S. C., Araújo, L. F., Levy, R. B., Barreto, S. M. & Giatti, L. Association between consumption of ultra-processed foods and serum C-reactive protein levels: cross-sectional results from the ELSA-Brasil study. *Sao Paulo Medical Journal* **137**, 169 – 176 (2019). URL http://www.scielo.br/scielo.php?script=sci_arttext&pid=S1516-31802019000200169&nrm=iso.

[53] Steele, E. M. & Monteiro, C. A. Association between dietary share of ultra-processed foods and urinary concentrations of phytoestrogens in the US. *Nutrients* **9** (2017).

[54] Gehring, J. *et al.* Consumption of Ultra-Processed Foods by Pesco-Vegetarians, Vegetarians, and Vegans: Associations with Duration and Age at Diet Initiation. *The Journal of Nutrition* **151**, 120–131 (2020). URL https://doi.org/10.1093/jn/nxaa196. https://academic.oup.com/jn/article-pdf/151/1/120/35365121/nxaa196.pdf.

[55] Buckley, J. P., Kim, H., Wong, E. & Rebholz, C. M. Ultra-processed food consumption and exposure to phthalates and bisphenols in the US National Health and Nutrition Examination Survey, 2013–2014. *Environment International* **131** (2019).

[56] Martínez Steele, E., Khandpur, N., da Costa Louzada, M. L. & Monteiro, C. A. Association between dietary contribution of ultra-processed foods and urinary concentrations of phthalates and bisphenol in a nationally representative sample of the us population aged 6 years and older. *PLOS ONE* **15**, 1–21 (2020). URL https://doi.org/10.1371/journal.pone.0236738.

[57] Morales, F. J., Mesías, M. & Delgado-Andrade, C. Association between heat-induced chemical markers and ultra-processed foods: A case study on breakfast cereals. *Nutrients* **12** (2020). URL https://www.mdpi.com/2072-6643/12/5/1418.

[58] Ibsen, D. B. *et al.* Food substitution models for nutritional epidemiology. *The American journal of clinical nutrition* **113**, 294–303 (2021).

[59] Adams, J., Hofman, K., Moubarac, J. C. & Thow, A. M. Public health response to ultra-processed food and drinks. *BMJ (Clinical research ed.)* **369**, m2391 (2020).

[60] What We Eat In America (WWEIA) Database. URL https://data.nal.usda.gov/dataset/what-we-eat-america-wweia-database.

[61] Open Food Facts - World (10/15/2021). URL https://world.openfoodfacts.org/.

[62] Formula to determine the Nova group (10/15/2021). URL https://world.openfoodfacts.org/nova.

[63] U.S. Department of Agriculture, A. R. S. FoodData Central: Foundation Foods (2019). URL fdc.nal.usda.gov.

[64] Dekker, M. & Verkerk, R. Dealing with variability in food production chains: A tool to enhance the sensitivity of epidemiological studies on phytochemicals. *European Journal of Nutrition* **42**, 67–72 (2003). URL https://link.springer.com/article/10.1007/s00394-003-0412-8.

[65] Ravandi, B., Mehler, P., Barabási, A.-L. & Menichetti, G. GroceryDB: A Database of Food and Beverage Products Annotated by Food Processing Characteristics in the US Grocery Stores (2021).

[66] FDA Center for Food Safety and Applied Nutrition. Guidance for Industry: Guide for Developing and Using Data Bases for Nutrition Labeling. URL https://www.fda.gov/regulatory-information/search-fda-guidance-documents/guidance-industry-guide-developing-and-using-data-bases-nutrition-labeling.

[67] Matthäus, B. & Haase, N. U. Acrylamide in ready-to-eat foods. In Kotzekidou, P. (ed.) *Food Hygiene and Toxicology in Ready-to-Eat Foods*, 353–382 (Academic Press, San Diego, 2016). URL https://www.sciencedirect.com/science/article/pii/B9780128019160000200.

[68] Phillips, D. H. Polycyclic aromatic hydrocarbons in the diet. *Mutation Research/Genetic Toxicology and Environmental Mutagenesis* **443**, 139–147 (1999). URL https://www.sciencedirect.com/science/article/pii/S1383574299000162.

[69] Schecter, A. *et al.* Polybrominated diphenyl ethers (pbdes) and hexabromocyclodecane (hbcd) in composite u.s. food samples. *Environmental Health Perspectives* **118**, 357–362 (2010). URL https://ehp.niehs.nih.gov/doi/abs/10.1289/ehp.0901345. https://ehp.niehs.nih.gov/doi/pdf/10.1289/ehp.0901345.

[70] Crews, C. & Castle, L. A review of the occurrence, formation and analysis of furan in heat-processed foods. *Trends in Food Science & Technology* **18**, 365–372 (2007). URL https://www.sciencedirect.com/science/article/pii/S0924224407000854.

[71] Ye, J., Wang, X.-H., Sang, Y.-X. & Liu, Q. Assessment of the determination of azodicarbonamide and its decomposition product semicarbazide: Investigation of variation in flour and flour products. *Journal of Agricultural and Food Chemistry* **59**, 9313–9318 (2011). URL https://doi.org/10.1021/jf201819x. PMID: 21786817, https://doi.org/10.1021/jf201819x.

[72] Willett, W. *Nutritional epidemiology* (1998).

[73] Reedy, J. *et al.* Evaluation of the healthy eating index-2015. *Journal of the Academy of Nutrition and Dietetics* **118**, 1622 – 1633 (2018). URL http://www.sciencedirect.com/science/article/pii/S2212267218308360.
