# Supplementary material for: Machine learning prediction of the degree of food processing
Source: Nat Commun. 2023 Apr 21;14:2312. doi: 10.1038/s41467-023-37457-1 (PMC10121643; doi:10.1038/s41467-023-37457-1)
Supplement: Supplementary file 3 — Reporting Summary [file 41467_2023_37457_MOESM3_ESM.pdf]

nature portfolio

Corresponding author(s): Albert-Laszlo Barabasi

Last updated by author(s): Mar 1, 2023

Reporting Summary

Nature Portfolio wishes to improve the reproducibility of the work that we publish. This form provides structure for consistency and transparency in reporting. For further information on Nature Portfolio policies, see our Editorial Policies and the Editorial Policy Checklist.

Statistics

For all statistical analyses, confirm that the following items are present in the figure legend, table legend, main text, or Methods section.

n/a	Confirmed	
☐	☒	The exact sample size (*n*) for each experimental group/condition, given as a discrete number and unit of measurement
☒	☐	A statement on whether measurements were taken from distinct samples or whether the same sample was measured repeatedly
☐	☒	The statistical test(s) used AND whether they are one- or two-sided<br>*Only common tests should be described solely by name; describe more complex techniques in the Methods section.*
☐	☒	A description of all covariates tested
☐	☒	A description of any assumptions or corrections, such as tests of normality and adjustment for multiple comparisons
☐	☒	A full description of the statistical parameters including central tendency (e.g. means) or other basic estimates (e.g. regression coefficient) AND variation (e.g. standard deviation) or associated estimates of uncertainty (e.g. confidence intervals)
☐	☒	For null hypothesis testing, the test statistic (e.g. *F*, *t*, *r*) with confidence intervals, effect sizes, degrees of freedom and *P* value noted<br>*Give P values as exact values whenever suitable.*
☒	☐	For Bayesian analysis, information on the choice of priors and Markov chain Monte Carlo settings
☒	☐	For hierarchical and complex designs, identification of the appropriate level for tests and full reporting of outcomes
☐	☒	Estimates of effect sizes (e.g. Cohen's *d*, Pearson's *r*), indicating how they were calculated

Our web collection on statistics for biologists contains articles on many of the points above.

Software and code

Policy information about availability of computer code

Data collection	No software was used for data collection.
Data analysis	The codes that support the findings of this study are openly available on our GitHub at https://doi.org/10.5281/zenodo.7700545 and https://github.com/menicgiulia/MLFoodProcessing. Python 3.6.10, MATLAB 2022a, and R (version 4.0.3 – 2020-10-10) were used for data analysis and visualization. The R Survey package (version 4.0) was used to incorporate survey weights in all analyses.

For manuscripts utilizing custom algorithms or software that are central to the research but not yet described in published literature, software must be made available to editors and reviewers. We strongly encourage code deposition in a community repository (e.g. GitHub). See the Nature Portfolio guidelines for submitting code & software for further information.

Data

Policy information about availability of data

All manuscripts must include a data availability statement. This statement should provide the following information, where applicable:

- Accession codes, unique identifiers, or web links for publicly available datasets
- A description of any restrictions on data availability
- For clinical datasets or third party data, please ensure that the statement adheres to our policy

The data generated and analyzed in the study have been deposited on Zenodo at https://doi.org/10.5281/zenodo.7700545. Detailed source data files are provided with this manuscript.
The publicly available datasets used in this study can be found on their associated websites:
FNDDS (https://www.ars.usda.gov/northeast-area/beltsville-md-bhnrc/beltsville-human-nutrition-research-center/food-surveys-research-group/docs/fndds-download-databases/),
NHANES (https://www.cdc.gov/nchs/nhanes/index.htm), NHANES exposome and phenome data (https://github.com/chiragjp/nhanes scidata), and Open Food Facts (https://world.openfoodfactdata)

Human research participants

Policy information about studies involving human research participants and Sex and Gender in Research.

Reporting on sex and gender	For the EWAS analysis we considered 20047 adults in NHANES 1999-2006, 18+ years old. This comprises both men (9562) and women (10485), with associated survey weights. We are not responsible for NHANES study design, as the National Health and Nutrition Examination Survey (NHANES) is a program of studies designed to assess the health and nutritional status of adults and children in the United States. NHANES is a major program publicly available surveys of the National Center for Health Statistics (NCHS), part of the Centers for Disease Control and Prevention (CDC) and responsible for producing vital and health statistics for the US. Our epidemiological analysis considers sex as a covariate and we mention this in the Results, in the Methods and in the Supplementary Materials. We also provide the analysis of the individual processing score iFPro stratified by sex and other covariates in Table S7 and S8.
Population characteristics	All models were adjusted for age, sex, ethnicity, Body Mass Index (BMI), total-caloric intake, and estimated Socioeconomic Status (SES)
Recruitment	We are not responsible for NHANES recruitment. See above.
Ethics oversight	We are not responsible for NHANES ethics oversight. See above.

Note that full information on the approval of the study protocol must also be provided in the manuscript.

Field-specific reporting

Please select the one below that is the best fit for your research. If you are not sure, read the appropriate sections before making your selection.

☒ Life sciences ☐ Behavioural & social sciences ☐ Ecological, evolutionary & environmental sciences

For a reference copy of the document with all sections, see nature.com/documents/nr-reporting-summary-flat.pdf

Life sciences study design

All studies must disclose on these points even when the disclosure is negative.

Sample size	The sample size regarding the machine learning algorithm FoodProX and FPro is determined by the food composition data currently available (foer example, for FNDDS 2009-2010 a total of 7,253 foods with 99 gram-based nutrients), and the amount of annotated foods by NOVA (2,484). For the EWAS analysis we considered 20047 adults in NHANES 1999-2006, 18+ years old. This a standard selection in nutritional epidemiological analysis. We are not responsible for NHANES study design, as the National Health and Nutrition Examination Survey (NHANES) is a program of studies designed to assess the health and nutritional status of adults and children in the United States. NHANES is a major program publicly available surveys of the National Center for Health Statistics (NCHS), part of the Centers for Disease Control and Prevention (CDC) and responsible for producing vital and health statistics for the US.
Data exclusions	For the model training there was no data exclusion. For the EWAS analysis we considered 20047 adults in NHANES 1999-2006, 18+ years old.
Replication	We tested the predicted NOVA classes and the Food Processing Score on 6 cycles of FNDDS and Open Food Facts , with different nutrient panels. The replication was independent and successful from a machine learning perspective (5-fold stratified cross validation with stable high performance). The epidemiological associations were validated with a literature search of independent studies (Supplementary Table 9).
Randomization	Traditional randomization strategies on cohorts/experiments do not apply to this paper. FPro is a machine learning -based score that

Randomization	measures the degree of processing of any food, and this is the focus of the paper. We further tested its predictive power in a environment-wide association study on NHANES, a cross-sectional study that is designed by the CDC.
Blinding	Traditional blinding strategies on cohorts/experiments do not apply to this paper. FPro is a machine learning -based score that measures the degree of processing of any food, and this is the focus of the paper. We further tested its predictive power in a environment-wide association study on NHANES, a cross-sectional study that is designed by the CDC.

Reporting for specific materials, systems and methods

We require information from authors about some types of materials, experimental systems and methods used in many studies. Here, indicate whether each material, system or method listed is relevant to your study. If you are not sure if a list item applies to your research, read the appropriate section before selecting a response.

Materials & experimental systems

n/a	Involved in the study
☒	☐ Antibodies
☒	☐ Eukaryotic cell lines
☒	☐ Palaeontology and archaeology
☒	☐ Animals and other organisms
☒	☐ Clinical data
☒	☐ Dual use research of concern

Methods

n/a	Involved in the study
☒	☐ ChIP-seq
☒	☐ Flow cytometry
☒	☐ MRI-based neuroimaging
